# Supplementary material for: Causal Machine Learning Methods and Use of Cross‐Fitting in Settings With High‐Dimensional Confounding
Source: Stat Med. 2025 Sep 23;44(20-22):e70272. doi: 10.1002/sim.70272 (PMC12457817; doi:10.1002/sim.70272)
Supplement: Supplementary file 1 — Data S1: Supporting Information. [file SIM-44-0-s001.pdf]

SUPPLEMENTARY MATERIAL: Causal machine learning methods and use of cross-fitting in settings with high-dimensional confounding

Section 1 | FURTHER DETAILS AND SUMMARY OF MOTIVATING EXAMPLE

TABLE S1: Characteristics of participants in the BIS inception birth cohort (n=1,074).

Characteristic	Inception birth cohort[1,#]	Missing data (%)^
Pre-pregnancy BMI (kg/m^2)	24.0 [21.5, 27.9]	149 (14%)
Socio-Economic Indexes for Areas (SEIFA)		3 (0.3%)
Low	357 (33%)	
Med	357 (33%)	
High	357 (33%)	
Mother smoking in pregnancy	169 (16%)	13 (1.2%)
Gestational diabetes in pregnancy	44 (4.8%)	166 (15%)
Pre-eclampsia in pregnancy	35 (3.3%)	4 (0.4%)
Birthweight (grams)	3,527 (519)	0 (0%)
Infant sex		0 (0%)
Female	519 (48%)	
Male	555 (52%)	
Maternal age at birth (years)	32.1 (4.8)	0 (0%)
Gestational age at birth		0 (0%)
32-36 completed weeks	47 (4.4%)	
37-42 completed weeks	1,027 (96%)	
Mode of birth		2 (0.2%)
Caesarean	333 (31%)	
Vaginal	739 (69%)	
Weight-for-length z-score at 12 months	0.72 (1.04)	214 (20%)
Age at 12-month measures (months)	13.03 (0.82)	194 (18%)
Breastfeeding duration (exclusive weeks*)	4 [1, 22]	295 (27%)
Postnatal smoke exposure	130 (15%)	197 (18%)
Inflammation (GlycA) at 1-year of age (mmol/l)	1.32 (0.22)	332 (31%)
Pulse wave velocity at 4-years of age (m/s)	3.97 (0.45)	486 (45%)

[1]Mean (SD), Median [IQR] or Frequency (%) as appropriate, [#]n=1074, ^Indicates % of n=1074,
*Number of weeks that infant was exclusively breastfed (i.e. no supplementary feeding)
GlycA: Glycoprotein Acetyls

Section 2 | DOUBLY ROBUST METHODS, IMPLEMENTATION WITH CROSS-FITTING

In this study, when CF was applied, point and variance estimates were obtained in the following manner for each DR method using different libraries in SL to fit the models.

Assume a dataset of dimension [n x p], where n refers to the number of observations (or rows) and p refers to the number of analysis variables. Let I_k denote the observations (a vector of indices) in fold k ($k = 1, ..., K$) , and I_k^C denote the indices corresponding to observations not in I_k. For fold k, outcome and exposure models are fit on I_k^C, and using these, predicted outcome and exposure values are obtained for those observations in I_k. We denote predicted values for outcome for fold k under $X = x$ as $\hat{E}_x^{(-k(i))}(W_i)$, and for exposure as $\hat{P}^{(-k(i))}(W_i)$, to indicate that predicted values for an individual i in fold k are obtained by using nuisance models that were fit on the complement of fold k (I_k^C). For AIPW with CF, the estimate of the ACE is calculated using,

$$\hat{\psi}_{\mathrm{AIPW}_{\mathrm{CF}}} = \frac{1}{n}\sum_{i=1}^{n}\left[\hat{E}_1^{(-k(i))}(W_i) - \hat{E}_0^{(-k(i))}(W_i) + X_i\frac{Y_i - \hat{E}_1^{(-k(i))}(W_i)}{\hat{P}^{(-k(i))}(W_i)} - (1 - X_i)\frac{Y_i - \hat{E}_0^{(-k(i))}(W_i)}{1 - \hat{P}^{(-k(i))}(W_i)}\right] \tag{S1}$$

and the variance is calculated for the ACE using,

$$\widehat{var}(\hat{\psi}_{AIPW_{CF}}) = \frac{1}{n-1}\sum_{i=1}^{n}\left[\hat{E}_1^{(-k(i))}(W_i) - \hat{E}_0^{(-k(i))}(W_i) + X_i\frac{Y_i - \hat{E}_1^{(-k(i))}(W_i)}{\hat{P}^{(-k(i))}(W_i)} - (1 - X_i)\frac{Y_i - \hat{E}_0^{(-k(i))}(W_i)}{1 - \hat{P}^{(-k(i))}(W_i)} - \hat{\psi}_{AIPW_{CF}}\right]^2 \tag{S2}$$

For TMLE with CF the estimate of the ACE is calculated using,

$$\hat{\psi}_{\mathrm{TMLE}_{CF}} = \frac{1}{n}\left[\sum_{i=1}^{n}\hat{E}_1^{*(-k(i))}(W_i) - \sum_{i=1}^{n}\hat{E}_0^{*(-k(i))}(W_i)\right] \tag{S3}$$

Similarly to AIPW, the variance for TMLE is as before (S2) but with $\hat{E}_1^{*(-k(i))}(W)$ and $\hat{E}_0^{*(-k(i))}(W)$ in place of $\hat{E}_0^{(-k(i))}(W)$ and $\hat{E}_0^{(-k(i))}(W)$ respectively.

Section 3 | FURTHER DETAILS REGARDING CONFOUNDERS

TABLE S2: Description and summary of background and demographic confounders in the motivating case study sample (n=252)

**Background/demographic**	**Confounder**	**Summary statistics**[1]
**Antenatal**	Pre pregnancy BMI (kg/m^2)	24.3 [21.7, 27.9]
	Socio-Economic Indexes for Areas (SEIFA)	
	Low	73 (29%)
	Med	93 (37%)
	High	86 (34%)
	Mother smoking in pregnancy	32 (13%)
	Gestational diabetes in pregnancy	11 (4.4%)
	Pre-eclampsia in pregnancy	9 (3.6%)
**Birth**	Birthweight (grams)	3,508 (537)
	Infant sex	
	Female	119 (47%)
	Male	133 (53%)
	Maternal age at birth (years)	32.3 (4.2)
	Gestational age at birth	
	32-36 completed weeks	13 (5.2%)
	37-42 completed weeks	239 (95%)
	Mode of birth	
	Caesarean	95 (38%)
	Vaginal	157 (62%)
**1 year**	Weight-for-length z-score at 12 months	0.72 (1.08)
	Age at 12-month measures (months)	12.94 (0.79)
	Breastfeeding duration (exclusive weeks)	7 [0, 22]
	Postnatal smoke exposure	39 (15%)

[1] Mean (SD), Median [IQR] or Frequency (%) as appropriate

TABLE S3: Description and summary of metabolite confounders

Metabolite*	Confounder	Summary statistics[1]
	Total lipids in chylomicrons and extremely large VLDL (mmol/l)	-4.44 (-8.11, -3.76)
	Total lipids in very large VLDL (mmol/l)	-3.31 (-5.74, -2.77)
	Total lipids in large VLDL (mmol/l)	-1.66 (-2.19, -1.29)
	Total lipids in medium VLDL (mmol/l)	-0.80 (-1.10, -0.55)
	Total lipids in small VLDL (mmol/l)	-0.83 (-1.00, -0.60)
	Total lipids in very small VLDL (mmol/l)	-1.02 (-1.15, -0.86)
	Total lipids in IDL (mmol/l)	-0.20 (-0.38, -0.07)
	Total lipids in large LDL (mmol/l)	-0.06 (-0.26, 0.07)
	Total lipids in medium LDL (mmol/l)	-0.61 (-0.81, -0.46)
	Total lipids in small LDL (mmol/l)	-1.03 (-1.19, -0.88)
	Total lipids in very large HDL (mmol/l)	-0.96 (-1.24, -0.72)
	Total lipids in large HDL (mmol/l)	-0.61 (-0.87, -0.36)
	Total lipids in medium HDL (mmol/l)	-0.33 (-0.44, -0.23)
	Total lipids in small HDL (mmol/l)	0.01 (-0.05, 0.06)
	Mean diameter for VLDL particles (nm)	3.61 (3.58, 3.63)
	Mean diameter for LDL particles (nm)	3.158 (3.155, 3.161)
	Mean diameter for HDL particles (nm)	2.289 (2.276, 2.303)
	Serum total cholesterol (mmol/l)	1.19 (1.04, 1.31)
	Total cholesterol in VLDL (mmol/l)	-0.84 (-1.09, -0.61)
	Remnant cholesterol (non-HDL, non-LDL -cholesterol) (mmol/l)	-0.10 (-0.28, 0.08)
	Total cholesterol in LDL (mmol/l)	0.14 (-0.10, 0.31)
	Total cholesterol in HDL (mmol/l)	0.18 (0.06, 0.29)
	Total cholesterol in HDL2 (mmol/l)	-0.31 (-0.48, -0.15)
	Total cholesterol in HDL3 (mmol/l)	-0.78 (-0.81, -0.74)
**1 year**	Esterified cholesterol (mmol/l)	0.83 (0.68, 0.96)
	Free cholesterol (mmol/l)	0.00 (-0.13, 0.11)
	Serum total triglycerides (mmol/l)	0.08 (-0.13, 0.28)
	Triglycerides in VLDL (mmol/l)	-0.33 (-0.63, -0.08)
	Triglycerides in LDL (mmol/l)	-1.86 (-2.04, -1.72)
	Triglycerides in HDL (mmol/l)	-2.17 (-2.35, -2.01)
	Total phosphoglycerides (mmol/l)	0.41 (0.32, 0.49)
	Ratio of triglycerides to phosphoglycerides	-0.53 (-0.74, -0.32)
	Phosphatidylcholine and other cholines (mmol/l)	0.45 (0.38, 0.54)
	Sphingomyelins (mmol/l)	-1.14 (-1.24, -1.02)
	Total cholines (mmol/l)	0.63 (0.54, 0.71)
	Apolipoprotein A-I (g/l)	0.25 (0.19, 0.31)
	Apolipoprotein B (g/l)	-0.39 (-0.52, -0.27)
	Ratio of apolipoprotein B to apolipoprotein A-I	-0.63 (-0.77, -0.52)
	Total fatty acids (mmol/l)	2.17 (2.06, 2.29)
	Estimated degree of unsaturation	0.16 (0.13, 0.19)
	22:6, docosahexaenoic acid (mmol/l)	-2.66 (-2.96, -2.44)
	18:2, linoleic acid (mmol/l)	0.79 (0.65, 0.89)
	Omega-3 fatty acids (mmol/l)	-1.29 (-1.49, -1.10)
	Omega-6 fatty acids (mmol/l)	0.99 (0.88, 1.09)
	Polyunsaturated fatty acids (mmol/l)	1.09 (0.97, 1.20)
	Monounsaturated fatty acids; 16:1, 18:1 (mmol/l)	0.95 (0.82, 1.09)
	Saturated fatty acids (mmol/l)	1.16 (1.06, 1.26)

Table S3: Description and summary of metabolite confounders (continued)

Metabolite*	Confounder	Summary statistics[1]
	Ratio of 22:6 docosahexaenoic acid to total fatty acids (%)	-0.22 (-0.48, -0.05)
	Ratio of 18:2 linoleic acid to total fatty acids (%)	3.21 (3.14, 3.28)
	Ratio of omega-3 fatty acids to total fatty acids (%)	1.15 (1.00, 1.27)
	Ratio of omega-6 fatty acids to total fatty acids (%)	3.42 (3.37, 3.47)
	Ratio of polyunsaturated fatty acids to total fatty acids (%)	3.53 (3.47, 3.57)
	Ratio of monounsaturated fatty acids to total fatty acids (%)	3.38 (3.33, 3.44)
	Ratio of saturated fatty acids to total fatty acids (%)	3.60 (3.56, 3.64)
	Glucose (mmol/l)	1.16 (0.80, 1.24)
	Lactate (mmol/l)	0.71 (0.47, 1.34)
	Pyruvate (mmol/l)	-1.94 (-2.17, -1.60)
	Citrate (mmol/l)	-2.11 (-2.19, -2.02)
**1 year**	Glycerol (mmol/l)	-2.27 (-2.44, -2.06)
	Alanine (mmol/l)	-1.03 (-1.16, -0.88)
	Glutamine (mmol/l)	-0.84 (-0.90, -0.75)
	Glycine (mmol/l)	-1.49 (-1.60, -1.37)
	Histidine (mmol/l)	-2.75 (-2.89, -2.60)
	Isoleucine (mmol/l)	-2.61 (-2.84, -2.42)
	Leucine (mmol/l)	-2.40 (-2.66, -2.20)
	Valine (mmol/l)	-1.57 (-1.81, -1.35)
	Phenylalanine (mmol/l)	-2.69 (-2.83, -2.60)
	Tyrosine (mmol/l)	-2.61 (-2.86, -2.42)
	Acetate (mmol/l)	-3.31 (-3.50, -3.03)
	Acetoacetate (mmol/l)	-2.77 (-3.22, -2.47)
	3-hydroxybutyrate (mmol/l)	-1.90 (-2.10, -1.65)
	Albumin (signal area)	-2.46 (-2.51, -2.41)
	Creatinine (mmol/l)	-3.95 (-4.08, -3.82)

*Log transformed (zero converted to half minimum (non-zero) detected prior to transformation)
[1]Mean (95% CI)

TABLE S4: Confounders included in each confounder set considered

Confounder set	Confounders included
Small (1)	All confounders listed in Table S2.
Large (2)	All confounders listed in Table S2 and Table S3.

Section 4 | DETAILS REGARDING DATA-GENERATION MECHANISMS

TABLE S5: Details (and order) of data generation for the confounders

Confounder	BIS variable	Generating distribution and details
C_1	Socio-Economic Indexes for Areas (SEIFA)	$C_1 \sim \text{Categorical}(\boldsymbol{\theta} = \theta_1, \theta_2, \theta_3)$
C_2	Pre-pregnancy BMI (kg/m^2)	$C_2 \sim \text{Lognormal}\left(log\left(\frac{\tau^2}{\sqrt{\rho^2+\tau^2}}\right), \sqrt{\log\left(\frac{\rho^2}{\tau^2}+1\right)}\right)$<br>$log(C_2) \sim \text{Normal}(\tau, \rho)$<br>$\tau : E(log(C_2)) = \beta_0 + \beta_1 C_{1,low} + \beta_2 C_{1,med}$
C_3	Mother smoking in pregnancy	$C_3 \sim \text{Binomial}(1, expit(\beta_0 + \beta_1 C_{1,low} + \beta_2 C_{1,med}))$
C_4	Gestational diabetes in pregnancy	$C_4 \sim \text{Binomial}(1, expit(\beta_0 + \beta_1 C_2))$
C_5	Pre-eclampsia in pregnancy	$C_5 \sim \text{Binomial}(1, expit(\beta_0 + \beta_1 C_2 + \beta_2 C_3))$
C_6	Infant sex	$C_6 \sim \text{Binomial}(1, p = 0.53)$
C_7	Maternal age at birth (years)	$C_7 = \text{Normal}(E(C_7)) = \beta_0 + \beta_1 C_{1,low} + \beta_2 C_{1,med}, \sigma = s_{C_7})$
C_8	Gestational age at birth (weeks)	$C_8 \sim \text{Binomial}(1, expit(\beta_0 + \beta_1 C_2 + \beta_2 C_3 + \beta_3 C_4 + \beta_4 C_5 + \beta_5 C_7))$
C_9	Mode of birth	$C_9 \sim \text{Binomial}(1, expit(\beta_0 + \beta_1 C_2 + \beta_s C_3 + \beta_3 C_4 + \beta_4 C_5 + \beta_5 C_7 + \beta_6 C_8))$
C_{10}	Birthweight (grams)	$C_{10} \sim \text{Normal}(E(C_{10}) = \beta_0 + \beta_1 C_2 + \beta_2 C_3 + \beta_3 C_4 + \beta_4 C_5 + \beta_6 C_7 +$<br>$\beta_7 C_8, \sigma = s_{C_10})$
C_{11}	Age at 12-month measures (months)	$C_{11} \sim \text{Lognormal}\left(log\left(\frac{\tau^2}{\sqrt{(\rho^2+\tau^2)}}\right), \sqrt{log\left(\frac{\rho^2}{\tau^2}+1\right)}\right)$<br>$log(C_{11}) \sim \text{Normal}(\tau, \rho)$
C_{12}	Breastfeeding duration (exclusive weeks)	$C_{12} \sim$ (zero-inflated) Poisson(μ, π)<br>$log(\mu) = \beta_0 + \beta_1 C_{1,low} + \beta_2 C_{1,med} + \beta_3 C_7 + \beta_4 C_8$ [counts]<br>$log(\pi) = \varphi_0 + \varphi_1 C_{1,low} + \varphi_2 C_{1,med} + \varphi_3 C_7 + \varphi_4 C_8$ [zero-inflation]
C_{13}	Postnatal smoke exposure	$C_{13} \sim \text{Binomial}(1, expit(\beta_0 + \beta_1 C_{1,low} + \beta_2 C_{1,med} + \beta_3 C_3)$
C_{14}	Weight-for-length z-score at 12 months	$C_{14} \sim \text{Normal}(E(C_{14} = \beta_0 + \beta_1 C_6 + \beta_2 C_{10} + \beta_3 C_{11} + \beta_4 C_{12}, \sigma = s_{C_{14}})$
$\boldsymbol{M} = M_1, ., M_{73}$	Metabolites	$\boldsymbol{M} \sim \text{MVN}[\boldsymbol{m}, \boldsymbol{\Sigma}]$<br>$\boldsymbol{M} = (M_1, ..., M_{73})^T$<br>$\boldsymbol{m} = (E(M_1), ..., E(M_{73}))^T$<br>$\Sigma_{i,j} = Cov(M_i, M_j)$<br>$1 \leq i,j \leq 73$

TABLE S6: Details (and order) of data generation for the exposure

Confounder set	Mechanism	Generating distribution and details
Small (1)	Simple-1	$X \sim \mathrm{N}\left(\gamma_0 + \sum_{j=1}^{15}(\gamma_j C_j), sd_x\right)$ with X then dichotomised using the 75th percentile.
Small (1)	Complex-1a	$X \sim \mathrm{N}(\gamma_0 + \sum_{j=1}^{15}(\gamma_j C_j) + \sum_{1\leq i<j\leq 14}\gamma_{(13+i+j)}C_iC_j, sd_x)$ $\Rightarrow$ but set coefficient terms for the interaction terms to **two** times that observed in the BIS data: $\gamma_{(13+i+j)} \rightarrow 2\gamma_{(13+i+j)}$ with X then dichotomised using the 75th percentile.
Small (1)	Complex-1b	$X \sim \mathrm{N}(\gamma_0 + \sum_{j=1}^{15}(\gamma_j C_j) + \sum_{1\leq i<j\leq 14}\gamma_{(13+i+j)}C_iC_j, sd_x)$ $\Rightarrow$ but set coefficient terms for the interaction terms to **four** times that observed in the BIS data: $\gamma_{(13+i+j)} \rightarrow 4\gamma_{(13+i+j)}$ with X then dichotomised using the 75th percentile.
Large (2)	Simple-2	$X \sim \mathrm{N}\left(\gamma_0 + \sum_{j=1}^{88}(\gamma_j C_j), sd_x\right)$ with X then dichotomised using the 75th percentile.
Large (2)	Complex-2	$X \sim \mathrm{N}\left(\gamma_0 + \sum_{j=1}^{88}(\gamma_j C_j) + (a), sd_x\right)$ Where (a) is interaction and squared terms as detailed in Tables S8 and S9, with X then dichotomised using the 75th percentile.

Note: For Small confounder set (1), C_j refers to background and demographic confounder j, ($j = 1, \ldots, 14$) and for Large confounder set (2), C_j refers to background, demographic and metabolite confounders j, ($j = 1, \ldots, 87$) as outlined in Table S4. $\gamma_0, \gamma_1, \ldots\gamma_{14}, \ldots, sd_x$ are parameter values, estimated using the Barwon Infant Study (BIS) data.

TABLE S7: Details (and order) of data generation for the outcome

Confounder set	Mechanism	Generating distribution and details
Small (1)	Simple-1	$Y \sim \mathrm{N}\left(\alpha_0 + \alpha_1 X + \sum_{j=2}^{16} \alpha_j C_{j-1}, sd_y = 1\right)$
Small (1)	Complex-1a	$Y \sim \mathrm{N}(\alpha_0 + \alpha_1 X + \sum_{j=2}^{16} \alpha_j C_{j-1} + \sum_{1 \leq i < j \leq 14} \alpha_{(14+i+j)} C_i C_j +$ $\alpha_{79} C_{10}^2 + \alpha_{80} C_{12}^2 + \alpha_{81} C_{14}^2 + \sum_{j=1}^{14} \alpha_{(82+j)} C_j X, sd_y = 1)$<br><br>⇒ but set coefficient terms for the interaction terms to **two** times that observed in the BIS data:<br><br>$\alpha_{(14+i+j)} \rightarrow 2\alpha_{(14+i+j)}$ and $\alpha_{(82+j)} \rightarrow 2\alpha_{(82+j)}$
Small (1)	Complex-1b	$Y \sim \mathrm{N}(\alpha_0 + \alpha_1 X + \sum_{j=2}^{16} \alpha_j C_{j-1} + \sum_{1 \leq i < j \leq 14} \alpha_{(14+i+j)} C_i C_j +$ $\alpha_{79} C_{10}^2 + \alpha_{80} C_{12}^2 + \alpha_{81} C_{14}^2 + \sum_{j=1}^{14} \alpha_{(82+j)} C_j X, sd_y = 1)$<br><br>⇒ but set coefficient terms for the interaction terms to **four** times that observed in the BIS data:<br><br>$\alpha_{(14+i+j)} \rightarrow 4\alpha_{(14+i+j)}$ and $\alpha_{(82+j)} \rightarrow 4\alpha_{(82+j)}$
Large (2)	Simple-2	$Y \sim \mathrm{N}\left(\alpha_0 + \alpha_1 X + \sum_{j=2}^{89} \alpha_j C_{j-1}, sd_y = 1\right)$
Large (2)	Complex-2	$Y \sim \mathrm{N}\left(\alpha_0 + \alpha_1 X + \sum_{j=2}^{89} \alpha_j C_{j-1} + (b), sd_y = 1\right)$<br>Where (b) is interaction and squared terms as detailed in Tables S10 and S11

Note: For Small confounder set (1), C_j refers to background and demographic confounder j, ($j = 1, \ldots, 14$) and for Large confounder set (2), C_j refers to background, demographic and metabolite confounders j, ($j = 1, \ldots, 87$) as outlined in Table S4. $\alpha_0, \alpha_1, ...\alpha_{15}, ...$ are parameter values, estimated using the Barwon Infant Study (BIS) data.

TABLE S8 Interaction and squared terms for the data-generation of exposure for Complex-2 (a)

	Background/demographic confounders													
Background/demographic/metabolite confounders	C_1	C_2	C_3	C_4	C_5	C_6	C_7	C_8	C_9	C_{10}	C_{11}	C_{12}	C_{13}	C_{14}
C_1		×	×			×	×		×		×	×	×	×
C_2		**×**				×	×		×	×	×	×	×	×
C_3						×	×		×	×	×	×	×	×
C_4														
C_5														
C_6							×		×		×	×	×	×
C_7									×	×	×	×	×	×
C_8														
C_9										×	×	×	×	×
C_{10}										**×**	×	×	×	×
C_{11}												×	×	×
C_{12}												**×**	×	×
C_{13}														×
C_{14}														**×**
M_1									×					
M_2									×					
M_4								×						
M_5									×					
M_6									×					
M_{11}									×					
M_{12}										×				
M_{15}									×					
M_{24}											×			
M_{29}											×			
M_{30}		×							×					
M_{32}											×			
M_{56}											×			
M_{57}							×							
M_{60}						×								
M_{70}		×												
M_{71}														×

Note: × indicates that interaction or squared term (bold) included.

TABLE S9 Interaction and squared terms for the data-generation of exposure for Complex-2 (a) (continued)

[**Note:** × indicates that interaction or squared term (bold) included.]

Metabolite confounders (columns); Metabolite confounders (rows)

	M_1	M_2	M_3	M_4	M_6	M_8	M_9	M_{10}	M_{11}	M_{12}	M_{14}	M_{16}	M_{17}	M_{22}	M_{23}	M_{24}	M_{30}	M_{31}	M_{33}	M_{35}	M_{36}	M_{37}	M_{41}	M_{42}	M_{46}	M_{47}	M_{50}	M_{52}	M_{56}	M_{57}	M_{60}	M_{61}	M_{62}	M_{71}
M_6					×																													
M_{11}		×	×																															
M_{12}										×																								
M_{14}	×	×	×	×																														
M_{17}	×	×	×	×																														
M_{18}						×	×	×																										
M_{22}									×																									
M_{23}									×																									
M_{25}							×	×																										
M_{26}						×	×	×																										
M_{30}																	**×**																	
M_{31}																		**×**																
M_{33}																			**×**															
M_{34}											×																							
M_{35}																				**×**														
M_{38}																						×												
M_{41}												×				×							**×**											
M_{42}																								**×**										
M_{45}																									×									
M_{46}																										×								
M_{48}												×				×																		
M_{50}																											**×**							
M_{53}																												×						
M_{55}																					×													
M_{56}																													**×**					
M_{57}																													×	**×**				
M_{58}	×																																	
M_{59}	×																				×													
M_{60}														×	×																			
M_{61}																																**×**		
M_{63}																															×			
M_{66}																																×		
M_{67}														×	×																			
M_{68}																												×				×	×	
M_{69}													×			×																		
M_{70}																													×	×				
M_{71}																														×	×			
M_{72}																																		×

Note: × indicates that interaction or squared term (bold) included.

TABLE S10 Interaction and squared terms for the data-generation of outcome for Complex-2 (b)

Background/demographic/metabolite confounders	Background/demographic confounders C_1	C_2	C_3	C_4	C_5	C_6	C_7	C_8	C_9	C_{10}	C_{11}	C_{12}	C_{13}	C_{14}	Exposure(X)
C_1		×	×			×	×		×	×	×	×	×	×	×
C_2			×			×	×		×	×	×	×	×	×	
C_3						×	×		×	×	×	×	×	×	×
C_6							×		×	×	×	×	×	×	×
C_7									×	×	×	×	×	×	
C_9										×	×	×	×	×	
C_{10}										**×**	×	×	×	×	
C_{11}												×	×	×	
C_{12}												**×**			×
C_{14}														**×**	
M_5			×												
M_6												×			
M_7			×				×					×			
M_8			×												
M_{14}							×								
M_{16}							×								
M_{19}												×			
M_{22}												×			
M_{24}									×						
M_{27}							×								
M_{29}							×								
M_{30}												×			
M_{32}										×					
M_{35}										×					
M_{43}										×					
M_{50}										×					
M_{57}										×					
M_{62}		×					×					×			
M_{64}										×					
M_{65}										×					
M_{66}							×								
M_{67}												×			
M_{69}									×	×					
M_{71}												×			

Note: × indicates that interaction or squared term (bold) included.

TABLE S11 Interaction and squared terms for the data-generation of outcome for Complex-2 (b) (continued)

Background/demographic/metabolite confounders	Background/demographic confounders										
	M_6	M_{10}	M_{18}	M_{38}	M_{48}	M_{64}	M_{65}	M_{66}	M_{68}	M_{70}	Exposure(X)
M_4											×
M_{10}											×
M_{11}		×									
M_{12}											×
M_{15}											×
M_{19}											×
M_{20}											×
M_{30}			×								
M_{34}		×									
M_{36}											×
M_{37}											×
M_{41}				×							
M_{42}			×	×							
M_{45}	×	×	×	×							
M_{47}	×	×	×								
M_{48}				×							
M_{49}			×	×	×						
M_{51}					×						
M_{52}					×						
M_{55}	×										×
M_{58}											×
M_{60}	×			×							×
M_{61}											×
M_{62}					×						×
M_{64}					×	**×**					
M_{65}							**×**				
M_{66}								**×**			
M_{67}		×									
M_{68}		×		×					**×**		
M_{70}						×	×	×	×	**×**	
M_{71}									×	×	
M_{73}	×	×									

TABLE S12 Parameter (coefficient) values used in the simulation study

Variable generated	Parameters	Values
C1	θ_1	0.333
	θ_2	0.333
	θ_3	0.333
C2	β_0 (Intercept)	3.237
	β_1	-0.004
	β_2	-0.056
	σ_{Logbmi}	0.198
C3	$\beta_0(Intercept)$	-2.001
	β_1	0.304
	β_2	-0.119
C4	β_0 (Intercept)	-6.907
	β_1	0.137
C5	β_0 (Intercept)	-4.409
	β_1	0.048
	β_2	-16.478
C6	p (Proportion male)	0.528
C7	β_0 (Intercept)	32.093
	β_1	0.080
	β_2	0.495
	σ_{matage}	4.187
C8	$\beta_0(Intercept)$	2.487
	β_1	0.014
	β_2	0.471
	β_3	-0.384
	β_4	-1.702
	β_5	0.006
C9	β_0 (Intercept)	3.960
	β_1	-0.022
	β_2	0.570
	β_3	-1.073
	β_4	-0.690
	β_5	-0.129
	β_6	1.379
C10	β_0 (Intercept)	2008.651
	β_1	22.017
	β_2	-224.591
	β_3	-171.003
	β_4	-518.665
	β_5	100.608
	β_6	1.625
	β_7	936.514
	$\sigma_{birthweight}$	464.864
C11	τ	2.559
	ρ	0.058
C12	β_0 (Intercept)	1.774
	β_1	-0.154
	β_2	0.174
	β_3	0.035
	ψ_0 (Intercept)	-1.246
	ψ_1	-0.384
	ψ_2	-0.005
	ψ_3	0.010
C13	β_0 (Intercept)	-1.789
	β_1	-0.424
	β_2	-0.985
	β_3	2.367
C14	β_0 (Intercept)	-3.247
	β_1	-0.128
	β_2	0.001
	β_3	0.160
	β_4	-0.008
	σ_{wflz}	1.026

TABLE S13 Parameter values (coefficients) used in the simulation study for the exposure (Simple-1 scenarios)

Parameters	Simple-1
γ_0	1.297
γ_1	-0.019
γ_2	-0.038
γ_3	-0.002
γ_4	0.021
γ_5	0.032
γ_6	-0.037
γ_7	-0.051
γ_8	0.005
γ_9	-0.043
γ_{10}	0.037
γ_{11}	0.0001
γ_{12}	-0.002
γ_{13}	-0.0001
γ_{14}	-0.042
γ_{15}	-0.003

TABLE S14 Parameter values (coefficients) used in the simulation study for the exposure (Complex-1a and Complex-1b scenarios)

Parameters	Complex-1a	Complex-1b	Parameters	Complex-1a	Complex-1b
γ_0	1.750	1.750	γ_{40}	0.0001	0.0001
γ_1	-0.998	-0.998	γ_{41}	0.006	0.013
γ_2	-1.098	-1.098	γ_{42}	0.001	0.002
γ_3	0.035	0.035	γ_{43}	-0.029	-0.058
γ_4	-1.983	-1.983	γ_{44}	-0.010	-0.020
γ_5	-0.089	-0.089	γ_{45}	-0.729	-1.458
γ_6	0.014	0.014	γ_{46}	0.067	0.135
γ_7	-0.083	-0.083	γ_{47}	-0.107	-0.215
γ_8	0.057	0.057	γ_{48}	0.0001	0.0001
γ_9	-0.181	-0.181	γ_{49}	0.153	0.305
γ_{10}	1.024	1.024	γ_{50}	-0.029	-0.059
γ_{11}	0.0001	0.0001	γ_{51}	-0.290	-0.580
γ_{12}	-0.091	-0.091	γ_{52}	-0.007	-0.015
γ_{13}	-0.045	-0.045	γ_{53}	0.003	0.007
γ_{14}	-0.609	-0.609	γ_{54}	-0.072	-0.145
γ_{15}	-0.267	-0.267	γ_{55}	0.0001	0.0001
			γ_{56}	0.042	0.085
γ_{16}	-0.008	-0.015	γ_{57}	-0.002	-0.004
γ_{17}	-0.011	-0.023	γ_{58}	0.411	0.823
γ_{18}	-0.528	-1.056	γ_{59}	-0.014	-0.029
γ_{19}	-0.874	-1.748	γ_{60}	0.004	0.009
γ_{20}	0.091	0.182	γ_{61}	0.0001	0.0001
γ_{21}	0.047	0.095	γ_{62}	-0.002	-0.005
γ_{22}	0.012	0.023	γ_{63}	0.0001	0.0001
γ_{23}	-0.040	-0.080	γ_{64}	-0.007	-0.015
γ_{24}	0.110	0.219	γ_{65}	-0.005	-0.010
γ_{25}	-0.047	-0.093	γ_{66}	0.0001	0.0001
γ_{26}	0.0001	0.0001	γ_{67}	-0.102	-0.203
γ_{27}	0.0001	0.001	γ_{68}	-0.001	-0.002
γ_{28}	0.105	0.210	γ_{69}	0.101	0.202
γ_{29}	0.222	0.445	γ_{70}	0.052	0.105
γ_{30}	0.001	0.002	γ_{71}	0.0001	0.0001
γ_{31}	-0.012	-0.024	γ_{72}	0.0001	0.0001
γ_{32}	0.629	1.259	γ_{73}	0.0001	0.0001
γ_{33}	0.290	0.579	γ_{74}	0.0001	0.0001
γ_{34}	0.165	0.329	γ_{75}	0.006	0.012
γ_{35}	-0.069	-0.138	γ_{76}	0.081	0.162
γ_{36}	0.025	0.050	γ_{77}	0.088	0.176
γ_{37}	-0.013	-0.026	γ_{78}	0.004	0.008
γ_{38}	-0.006	-0.013	γ_{79}	0.003	0.007
γ_{39}	-0.003	-0.006	γ_{80}	-0.012	-0.024

TABLE S15 Parameter values (coefficients) used in the simulation study for the exposure (Simple-2 scenarios)

Parameters	Simple-2	Parameters	Simple-2
γ_0	1.315	γ_{44}	-0.103
γ_1	0.038	γ_{45}	0.054
γ_2	0.035	γ_{46}	-0.342
γ_3	0.001	γ_{47}	-0.143
γ_4	-0.001	γ_{48}	0.423
γ_5	-0.036	γ_{49}	0.079
γ_6	0.040	γ_{50}	0.117
γ_7	-0.016	γ_{51}	1.198
γ_8	-0.003	γ_{52}	-1.898
γ_9	0.027	γ_{53}	1.540
γ_{10}	-0.0003	γ_{54}	-8.103
γ_{11}	0.0001	γ_{55}	-0.022
γ_{12}	0.002	γ_{56}	-0.780
γ_{13}	0.0005	γ_{57}	1.755
γ_{14}	0.005	γ_{58}	-4.55
γ_{15}	0.012	γ_{59}	7.848
		γ_{60}	-7.035
γ_{16}	-0.023	γ_{61}	-1.566
γ_{17}	0.014	γ_{62}	9.572
γ_{18}	-0.021	γ_{63}	0.790
γ_{19}	-0.243	γ_{64}	-1.338
γ_{20}	0.020	γ_{65}	3.400
γ_{21}	0.421	γ_{66}	-3.107
γ_{22}	-0.425	γ_{67}	3.091
γ_{23}	0.937	γ_{68}	0.726
γ_{24}	0.155	γ_{69}	-3.034
γ_{25}	-0.166	γ_{70}	-0.026
γ_{26}	0.004	γ_{71}	0.051
γ_{27}	0.038	γ_{72}	0.058
γ_{28}	-0.151	γ_{73}	-0.002
γ_{29}	-0.243	γ_{74}	0.032
γ_{30}	0.551	γ_{75}	-0.007
γ_{31}	-0.021	γ_{76}	0.013
γ_{32}	-0.404	γ_{77}	0.030
γ_{33}	3.978	γ_{78}	-0.034
γ_{34}	-0.193	γ_{79}	0.001
γ_{35}	0.218	γ_{80}	0.040
γ_{36}	-0.296	γ_{81}	-0.012
γ_{37}	-1.029	γ_{82}	0.035
γ_{38}	0.946	γ_{83}	0.002
γ_{39}	0.147	γ_{84}	0.024
γ_{40}	-3.534	γ_{85}	-0.017
γ_{41}	-1.129	γ_{86}	0.015
γ_{42}	0.388	γ_{87}	0.004
γ_{43}	-0.451	γ_{88}	0.008

TABLE S16 Parameter values (coefficients) used in the simulation study for the exposure (Complex-2 scenarios)

Parameters	Complex-2	Parameters	Complex-2
γ_0	5.885	γ_{57}	14.641
γ_1	0.238	γ_{58}	-9.326
γ_2	-0.595	γ_{59}	-14.181
γ_3	0.012	γ_{60}	18.410
γ_4	0.912	γ_{61}	-2.712
γ_5	0.002	γ_{62}	-7.694
γ_6	0.013	γ_{63}	-2.915
γ_7	0.561	γ_{64}	-8.897
γ_8	-0.061	γ_{65}	7.075
γ_9	-0.030	γ_{66}	8.473
γ_{10}	0.427	γ_{67}	-9.826
γ_{11}	-0.001	γ_{68}	1.187
γ_{12}	-0.323	γ_{69}	2.511
γ_{13}	0.0001	γ_{70}	0.029
γ_{14}	-0.846	γ_{71}	-0.332
γ_{15}	-0.775	γ_{72}	0.192
γ_{16}	-0.086	γ_{73}	-0.029
γ_{17}	0.135	γ_{74}	0.044
γ_{18}	0.020	γ_{75}	-0.065
γ_{19}	-0.441	γ_{76}	0.019
γ_{20}	0.066	γ_{77}	0.006
γ_{21}	-0.789	γ_{78}	0.007
γ_{22}	-0.621	γ_{79}	0.037
γ_{23}	-0.864	γ_{80}	-0.011
γ_{24}	-1.071	γ_{81}	0.001
γ_{25}	1.167	γ_{82}	0.004
γ_{26}	0.106	γ_{83}	-0.001
γ_{27}	-0.296	γ_{84}	0.009
γ_{28}	0.086	γ_{85}	-0.050
γ_{29}	-0.145	γ_{86}	0.028
γ_{30}	-0.910	γ_{87}	-0.022
γ_{31}	0.382	γ_{88}	0.020
γ_{32}	-0.051	γ_{89}	0.012
γ_{33}	15.641	γ_{90}	-0.028
γ_{34}	0.249	γ_{91}	0.006
γ_{35}	-1.150	γ_{92}	-0.022
γ_{36}	0.502	γ_{93}	-0.017
γ_{37}	-2.311	γ_{94}	0.022
γ_{38}	0.741	γ_{95}	0.072
γ_{39}	-0.275	γ_{96}	0.013
γ_{40}	-10.073	γ_{97}	0.039
γ_{41}	-3.425	γ_{98}	0.004
γ_{42}	0.372	γ_{99}	-0.060
γ_{43}	1.053	γ_{100}	-0.037
γ_{44}	0.110	γ_{101}	0.001
γ_{45}	-0.115	γ_{102}	0.122
γ_{46}	-0.127	γ_{103}	0.047
γ_{47}	-0.334	γ_{104}	-0.017
γ_{48}	0.126	γ_{105}	-0.020
γ_{49}	0.177	γ_{106}	-0.057
γ_{50}	0.298	γ_{107}	-0.189
γ_{51}	2.783	γ_{108}	-0.031
γ_{52}	-4.859	γ_{109}	0.087
γ_{53}	4.431	γ_{110}	-0.005
γ_{54}	-1.421	γ_{111}	-0.030
γ_{55}	-0.032	γ_{112}	0.080
γ_{56}	3.530	γ_{113}	0.009

TABLE S17 Parameter values (coefficients) used in the simulation study for the exposure (Complex-2 scenarios) (continued)

Parameters	Complex-2	Parameters	Complex-2
γ_{114}	0.253	γ_{174}	-0.061
γ_{115}	-0.312	γ_{175}	-0.001
γ_{116}	109.536	γ_{176}	-0.010
γ_{117}	-79.449	γ_{177}	-0.154
γ_{118}	-30.781	γ_{178}	-0.144
γ_{119}	-107.067	γ_{179}	0.0001
γ_{120}	77.438	γ_{180}	0.0001
γ_{121}	30.413	γ_{181}	-0.018
γ_{122}	0.103	γ_{182}	0.068
γ_{123}	-0.125	γ_{183}	0.001
γ_{124}	-0.049	γ_{184}	-0.0004
γ_{125}	0.069	γ_{185}	-0.212
γ_{126}	-0.043	γ_{186}	-0.206
γ_{127}	-0.004	γ_{187}	0.041
γ_{128}	0.064	γ_{188}	0.002
γ_{129}	0.047	γ_{189}	-0.004
γ_{130}	-0.019	γ_{190}	-0.013
γ_{131}	-0.091	γ_{191}	-0.0003
γ_{132}	0.056	γ_{192}	-0.002
γ_{133}	-0.099	γ_{193}	0.0001
γ_{134}	0.067	γ_{194}	0.0001
γ_{135}	-0.016	γ_{195}	0.0001
γ_{136}	0.012	γ_{196}	-0.004
γ_{137}	-0.277	γ_{197}	0.003
γ_{138}	-0.052	γ_{198}	-0.054
γ_{139}	0.007	γ_{199}	-0.012
γ_{140}	-0.014	γ_{200}	0.388
γ_{141}	0.192	γ_{201}	-0.0001
γ_{142}	-0.014	γ_{202}	-0.022
γ_{143}	-0.062	γ_{203}	0.008
γ_{144}	0.064	γ_{204}	-0.098
γ_{145}	-0.027	γ_{205}	0.082
γ_{146}	0.016	γ_{206}	-0.005
γ_{147}	0.028	γ_{207}	-0.013
γ_{148}	-0.035	γ_{208}	0.0001
γ_{149}	-0.006	γ_{209}	-0.003
γ_{150}	0.016	γ_{210}	0.002
γ_{151}	-0.053	γ_{211}	-0.078
γ_{152}	0.042	γ_{212}	0.017
γ_{153}	0.034	γ_{213}	-0.004
γ_{154}	-0.123	γ_{214}	0.0001
γ_{155}	0.063	γ_{215}	0.004
γ_{156}	-0.018	γ_{216}	-0.0001
γ_{157}	0.0001	γ_{217}	-0.006
γ_{158}	0.038	γ_{218}	0.008
γ_{159}	0.030	γ_{219}	0.0001
γ_{160}	-0.015	γ_{220}	-0.025
γ_{161}	0.002	γ_{221}	-0.002
γ_{162}	0.042	γ_{222}	-0.073
γ_{163}	0.035	γ_{223}	0.030
γ_{164}	0.030	γ_{224}	0.0001
γ_{165}	-0.002	γ_{225}	0.0001
γ_{166}	0.046	γ_{226}	0.0001
γ_{167}	0.003	γ_{227}	0.0001
γ_{168}	-0.003	γ_{228}	-0.001
γ_{169}	0.002	γ_{229}	0.109
γ_{170}	0.012	γ_{230}	0.026
γ_{171}	-0.004	γ_{231}	-0.006
γ_{172}	-0.062	γ_{232}	0.0004
γ_{173}	-0.068	γ_{233}	-0.117

TABLE S18 Parameter values (coefficients) used in the simulation study for the outcome (Simple-1 scenarios)

**Parameters**	**Values**			
	n=200	n=500	n=1000	n=2000
α_0	-0.670*	-0.670*	-0.670*	-0.670*
α_1	0.450	0.300	0.200	0.150
α_2	-0.159	-0.159	-0.159	-0.159
α_3	0.021	0.021	0.021	0.021
α_4	0.011	0.011	0.011	0.011
α_5	0.276	0.276	0.276	0.276
α_6	0.039	0.039	0.039	0.039
α_7	-0.071	-0.071	-0.071	-0.071
α_8	-0.109	-0.109	-0.109	-0.109
α_9	-0.019	-0.019	-0.019	-0.019
α_{10}	-0.056	-0.056	-0.056	-0.056
α_{11}	0.049	0.049	0.049	0.049
α_{12}	0.0001	0.0001	0.0001	0.0001
α_{13}	0.057	0.057	0.057	0.057
α_{14}	0.006	0.006	0.006	0.006
α_{15}	-0.007	-0.007	-0.007	-0.007
α_{16}	0.010	0.010	0.010	0.010

*Intercepts in all outcome models were modified so that the mean of Y remained as 0.

TABLE S19 Parameter values (coefficients) used in the simulation study for the outcome (Complex-1a and Complex-1b scenarios)

Parameters	Complex-1a				Complex-1b			
	n=200	n=500	n=1000	n=2000	n=200	n=500	n=1000	n=2000
α_0	-8.554*	-8.554*	-8.554*	-8.554*	-8.554*	-8.554*	-8.554*	-8.554*
α_1	-0.900	-1.250	-1.500	-1.650	-2.000	-2.750	-3.125	-3.425
α_2	1.436	1.436	1.436	1.436	1.436	1.436	1.436	1.436
α_3	2.596	2.596	2.596	2.596	2.596	2.596	2.596	2.596
α_4	-0.294	-0.294	-0.294	-0.294	-0.294	-0.294	-0.294	-0.294
α_5	2.247	2.247	2.247	2.247	2.247	2.247	2.247	2.247
α_6	-0.135	-0.135	-0.135	-0.135	-0.135	-0.135	-0.135	-0.135
α_7	-0.002	-0.002	-0.002	-0.002	-0.002	-0.002	-0.002	-0.002
α_8	0.057	0.057	0.057	0.057	0.057	0.057	0.057	0.057
α_9	0.213	0.213	0.213	0.213	0.213	0.213	0.213	0.213
α_{10}	0.002	0.002	0.002	0.002	0.002	0.002	0.002	0.002
α_{11}	1.141	1.141	1.141	1.141	1.141	1.141	1.141	1.141
α_{12}	0.002	0.002	0.002	0.002	0.002	0.002	0.002	0.002
α_{13}	0.984	0.984	0.984	0.984	0.984	0.984	0.984	0.984
α_{14}	-0.127	-0.127	-0.127	-0.127	-0.127	-0.127	-0.127	-0.127
α_{15}	-1.504	-1.504	-1.504	-1.504	-1.504	-1.504	-1.504	-1.504
α_{16}	-2.693	-2.693	-2.693	-2.693	-2.693	-2.693	-2.693	-2.693
α_{17}	0.0001	0.0001	0.0001	0.0001	0.0002	0.0002	0.0002	0.0002
α_{18}	0.0001	0.0001	0.0001	0.0001	0.0002	0.0002	0.0002	0.0002
α_{19}	-0.013	-0.013	-0.013	-0.013	-0.013	-0.013	-0.013	-0.013
α_{20}	-0.130	-0.130	-0.130	-0.130	-0.260	-0.260	-0.260	-0.260
α_{21}	0.009	0.009	0.009	0.009	0.017	0.017	0.017	0.017
α_{22}	1.930	1.930	1.930	1.930	3.860	3.860	3.860	3.860
α_{23}	0.925	0.925	0.925	0.925	1.850	1.850	1.850	1.850
α_{24}	0.236	0.236	0.236	0.236	0.472	0.472	0.472	0.472
α_{25}	0.377	0.377	0.377	0.377	0.754	0.754	0.754	0.754
α_{26}	0.141	0.141	0.141	0.141	0.282	0.282	0.282	0.282
α_{27}	0.046	0.046	0.046	0.046	0.092	0.092	0.092	0.092
α_{28}	0.112	0.112	0.112	0.112	0.223	0.223	0.223	0.223
α_{29}	0.544	0.544	0.544	0.544	1.087	1.087	1.087	1.087
α_{30}	0.001	0.001	0.001	0.001	0.002	0.002	0.002	0.002
α_{31}	-0.001	-0.001	-0.001	-0.001	-0.002	-0.002	-0.002	-0.002
α_{32}	-0.718	-0.718	-0.718	-0.718	-1.437	-1.437	-1.437	-1.437
α_{33}	-0.356	-0.356	-0.356	-0.356	-0.712	-0.712	-0.712	-0.712
α_{34}	0.003	0.003	0.003	0.003	0.006	0.006	0.006	0.006
α_{35}	0.002	0.002	0.002	0.002	0.003	0.003	0.003	0.003
α_{36}	-0.004	-0.004	-0.004	-0.004	-0.007	-0.007	-0.007	-0.007
α_{37}	-0.843	-0.843	-0.843	-0.843	-1.686	-1.686	-1.686	-1.686
α_{38}	0.084	0.084	0.084	0.084	0.167	0.167	0.167	0.167
α_{39}	-0.095	-0.095	-0.095	-0.095	-0.191	-0.191	-0.191	-0.191
α_{40}	0.276	0.276	0.276	0.276	0.552	0.552	0.552	0.552
α_{41}	-0.045	-0.045	-0.045	-0.045	-0.090	-0.090	-0.090	-0.090
α_{42}	0.004	0.004	0.004	0.004	0.009	0.009	0.009	0.009
α_{43}	0.042	0.042	0.042	0.042	0.084	0.084	0.084	0.084
α_{44}	0.0001	0.0001	0.0001	0.0001	0.0002	0.0002	0.0002	0.0002
α_{45}	0.029	0.029	0.029	0.029	0.058	0.058	0.058	0.058
α_{46}	-0.003	-0.003	-0.003	-0.003	-0.006	-0.006	-0.006	-0.006
α_{47}	-0.034	-0.034	-0.034	-0.034	-0.067	-0.067	-0.067	-0.067
α_{48}	0.016	0.016	0.016	0.016	0.032	0.032	0.032	0.032
α_{49}	0.822	0.822	0.822	0.822	1.645	1.645	1.645	1.645
α_{50}	-0.257	-0.257	-0.257	-0.257	-0.515	-0.515	-0.515	-0.515

*Intercepts in all outcome models were modified so that the mean of Y remained as 0.

TABLE S20 Parameter values (coefficients) used in the simulation study for the outcome (Complex-1a and Complex-1b scenarios) (continued)

Parameters	Complex-1a				Complex-1b			
	n=200	n=500	n=1000	n=2000	n=200	n=500	n=1000	n=2000
α_{51}	0.029	0.029	0.029	0.029	0.058	0.058	0.058	0.058
α_{52}	-0.002	-0.002	-0.002	-0.002	-0.004	-0.004	-0.004	-0.004
α_{53}	0.149	0.149	0.149	0.149	0.297	0.297	0.297	0.297
α_{54}	0.056	0.056	0.056	0.056	0.112	0.112	0.112	0.112
α_{55}	-0.344	-0.344	-0.344	-0.344	-0.688	-0.688	-0.688	-0.688
α_{56}	-1.451	-1.451	-1.451	-1.451	-2.902	-2.902	-2.902	-2.902
α_{57}	-0.012	-0.012	-0.012	-0.012	-0.023	-0.023	-0.023	-0.023
α_{58}	-0.623	-0.623	-0.623	-0.623	-1.246	-1.246	-1.246	-1.246
α_{59}	0.001	0.001	0.001	0.001	0.002	0.002	0.002	0.002
α_{60}	-0.120	-0.120	-0.120	-0.120	-0.240	-0.240	-0.240	-0.240
α_{61}	0.002	0.002	0.002	0.002	0.003	0.003	0.003	0.003
α_{62}	-0.818	-0.818	-0.818	-0.818	-1.636	-1.636	-1.636	-1.636
α_{63}	-0.032	-0.032	-0.032	-0.032	-0.064	-0.064	-0.064	-0.064
α_{64}	-0.001	-0.001	-0.001	-0.001	-0.002	-0.002	-0.002	-0.002
α_{65}	0.0001	0.0001	0.0001	0.0001	0.0002	0.0002	0.0002	0.0002
α_{66}	-0.027	-0.027	-0.027	-0.027	-0.053	-0.053	-0.053	-0.053
α_{67}	-0.002	-0.002	-0.002	-0.002	-0.004	-0.004	-0.004	-0.004
α_{68}	0.120	0.120	0.120	0.120	0.240	0.240	0.240	0.240
α_{69}	-0.082	-0.082	-0.082	-0.082	-0.164	-0.164	-0.164	-0.164
α_{70}	0.0001	0.0001	0.0001	0.0001	0.0002	0.0002	0.0002	0.0002
α_{71}	-0.207	-0.207	-0.207	-0.207	-0.414	-0.414	-0.414	-0.414
α_{72}	-0.018	-0.018	-0.018	-0.018	-0.037	-0.037	-0.037	-0.037
α_{73}	0.675	0.675	0.675	0.675	1.351	1.351	1.351	1.351
α_{74}	-0.389	-0.389	-0.389	-0.389	-0.778	-0.778	-0.778	-0.778
α_{75}	0.0001	0.0001	0.0001	0.0001	0.0002	0.0002	0.0002	0.0002
α_{76}	-0.0005	-0.0005	-0.0005	-0.0005	-0.001	-0.001	-0.001	-0.001
α_{77}	0.001	0.001	0.001	0.001	0.002	0.002	0.002	0.002
α_{78}	0.0001	0.0001	0.0001	0.0001	0.0002	0.0002	0.0002	0.0002
α_{79}	0.032	0.032	0.032	0.032	0.063	0.063	0.063	0.063
α_{80}	-0.316	-0.316	-0.316	-0.316	-0.633	-0.633	-0.633	-0.633
α_{81}	0.580	0.580	0.580	0.580	1.160	1.160	1.160	1.160
α_{82}	0.057	0.057	0.057	0.057	0.114	0.114	0.114	0.114
α_{83}	0.012	0.012	0.012	0.012	0.024	0.024	0.024	0.024
α_{84}	0.162	0.162	0.162	0.162	0.324	0.324	0.324	0.324
α_{85}	1.310	1.310	1.310	1.310	2.620	2.620	2.620	2.620
α_{86}	1.675	1.675	1.675	1.675	3.349	3.349	3.349	3.349
α_{87}	0.113	0.113	0.113	0.113	0.226	0.226	0.226	0.226
α_{88}	1.973	1.973	1.973	1.973	3.945	3.945	3.945	3.945
α_{89}	-0.934	-0.934	-0.934	-0.934	-1.868	-1.868	-1.868	-1.868
α_{90}	0.189	0.189	0.189	0.189	0.378	0.378	0.378	0.378
α_{91}	-0.255	-0.255	-0.255	-0.255	-0.510	-0.510	-0.510	-0.510
α_{92}	-0.001	-0.001	-0.001	-0.001	-0.002	-0.002	-0.002	-0.002
α_{93}	-0.433	-0.433	-0.433	-0.433	-0.865	-0.865	-0.865	-0.865
α_{94}	0.082	0.082	0.082	0.082	0.165	0.165	0.165	0.165
α_{95}	-0.404	-0.404	-0.404	-0.404	-0.808	-0.808	-0.808	-0.808
α_{96}	0.355	0.355	0.355	0.355	0.710	0.710	0.710	0.710

T A B L E S21 Parameter values (coefficients) used in the simulation study for the outcome (Simple-2 scenarios)

Parameters	**Simple-2**				Parameters	**Simple-2**
	n=200	n=500	n=1000	n=2000		All n
α_0	-0.585*	-0.585*	-0.585*	-0.585*	α_{45}	0.714
α_1	-0.650	-0.400	-0.300	-0.180	α_{46}	-0.364
α_2	-0.131	-0.131	-0.131	-0.131	α_{47}	1.952
α_3	-0.089	-0.089	-0.089	-0.089	α_{48}	1.335
α_4	0.011	0.011	0.011	0.011	α_{49}	-3.255
α_5	0.161	0.161	0.161	0.161	α_{50}	-0.130
α_6	0.285	0.285	0.285	0.285	α_{51}	2.139
α_7	0.321	0.321	0.321	0.321	α_{52}	-29.682
α_8	-0.164	-0.164	-0.164	-0.164	α_{53}	56.226
α_9	-0.005	-0.005	-0.005	-0.005	α_{54}	-56.620
α_{10}	-0.203	-0.203	-0.203	-0.203	α_{55}	77.082
α_{11}	0.210	0.210	0.210	0.210	α_{56}	0.020
α_{12}	0.0001	0.0001	0.0001	0.0001	α_{57}	1.921
α_{13}	0.020	0.020	0.020	0.020	α_{58}	95.457
α_{14}	0.005	0.005	0.005	0.005	α_{59}	-104.288
α_{15}	0.151	0.151	0.151	0.151	α_{60}	-75.338
α_{16}	-0.006	-0.006	-0.006	-0.006	α_{61}	81.208
α_{17}	0.240	0.240	0.240	0.240	α_{62}	-54.754
α_{18}	-0.057	-0.057	-0.057	-0.057	α_{63}	-59.246
α_{19}	0.521	0.521	0.521	0.521	α_{64}	-1.987
α_{20}	-1.307	-1.307	-1.307	-1.307	α_{65}	-55.721
α_{21}	-0.510	-0.510	-0.510	-0.510	α_{66}	79.113
α_{22}	-4.193	-4.193	-4.193	-4.193	α_{67}	40.556
α_{23}	1.576	1.576	1.576	1.576	α_{68}	-43.555
α_{24}	-8.678	-8.678	-8.678	-8.678	α_{69}	20.110
α_{25}	4.183	4.183	4.183	4.183	α_{70}	18.066
α_{26}	-4.151	-4.151	-4.151	-4.151	α_{71}	-0.041
α_{27}	0.148	0.148	0.148	0.148	α_{72}	-0.069
α_{28}	0.035	0.035	0.035	0.035	α_{73}	0.866
α_{29}	0.977	0.977	0.977	0.977	α_{74}	0.238
α_{30}	-0.249	-0.249	-0.249	-0.249	α_{75}	-0.069
α_{31}	-3.932	-3.932	-3.932	-3.932	α_{76}	-0.215
α_{32}	0.574	0.574	0.574	0.574	α_{77}	0.119
α_{33}	-0.367	-0.367	-0.367	-0.367	α_{78}	0.106
α_{34}	-18.918	-18.918	-18.918	-18.918	α_{79}	0.100
α_{35}	0.851	0.851	0.851	0.851	α_{80}	-0.294
α_{36}	1.371	1.371	1.371	1.371	α_{81}	1.022
α_{37}	5.427	5.427	5.427	5.427	α_{82}	-0.352
α_{38}	-0.804	-0.804	-0.804	-0.804	α_{83}	-0.019
α_{39}	-0.277	-0.277	-0.277	-0.277	α_{84}	-0.042
α_{40}	0.371	0.371	0.371	0.371	α_{85}	0.021
α_{41}	13.497	13.497	13.497	13.497	α_{86}	-0.065
α_{42}	8.655	8.655	8.655	8.655	α_{87}	-0.104
α_{43}	-3.056	-3.056	-3.056	-3.056	α_{88}	-0.013
α_{44}	7.012	7.012	7.012	7.012	α_{89}	-0.050

*Intercepts in all outcome models were modified so that the mean of Y remained as 0.

TABLE S22 Parameter values (coefficients) used in the simulation study for the outcome (Complex-2 scenarios)

Parameters	**Complex-2**				Parameters	**Complex-2**	Parameters	**Complex-2**
	n=200	n=500	n=1000	n=2000		All n		All n
α_0	55.665*	55.665*	55.665*	55.665*	α_{43}	-8.769	α_{86}	-0.270
α_1	1.700	0.200	-1.400	-1.600	α_{44}	29.406	α_{87}	0.646
α_2	9.642	9.642	9.642	9.642	α_{45}	0.908	α_{88}	-0.534
α_3	-4.643	-4.643	-4.643	-4.643	α_{46}	0.336	α_{89}	-0.122
α_4	0.764	0.764	0.764	0.764	α_{47}	8.362	α_{90}	0.0001
α_5	2.521	2.521	2.521	2.521	α_{48}	3.739	α_{91}	-0.0002
α_6	0.454	0.454	0.454	0.454	α_{49}	-1.080	α_{92}	-0.121
α_7	3.355	3.355	3.355	3.355	α_{50}	0.449	α_{93}	-0.386
α_8	-4.917	-4.917	-4.917	-4.917	α_{51}	-11.492	α_{94}	-0.137
α_9	-1.211	-1.211	-1.211	-1.211	α_{52}	77.843	α_{95}	0.175
α_{10}	-1.952	-1.952	-1.952	-1.952	α_{53}	-141.213	α_{96}	0.407
α_{11}	9.045	9.045	9.045	9.045	α_{54}	145.671	α_{97}	0.558
α_{12}	-0.010	-0.010	-0.010	-0.010	α_{55}	9.983	α_{98}	-0.870
α_{13}	-4.966	-4.966	-4.966	-4.966	α_{56}	-1.103	α_{99}	-0.389
α_{14}	0.451	0.451	0.451	0.451	α_{57}	15.394	α_{100}	-0.250
α_{15}	-22.933	-22.933	-22.933	-22.933	α_{58}	561.710	α_{101}	0.665
α_{16}	-2.863	-2.863	-2.863	-2.863	α_{59}	-579.619	α_{102}	0.045
α_{17}	-0.016	-0.016	-0.016	-0.016	α_{60}	-244.248	α_{103}	0.674
α_{18}	1.584	1.584	1.584	1.584	α_{61}	134.083	α_{104}	-0.450
α_{19}	1.465	1.465	1.465	1.465	α_{62}	42.367	α_{105}	3.542
α_{20}	-15.020	-15.020	-15.020	-15.020	α_{63}	-73.747	α_{106}	-4.306
α_{21}	-0.803	-0.803	-0.803	-0.803	α_{64}	-13.347	α_{107}	1.092
α_{22}	-13.349	-13.349	-13.349	-13.349	α_{65}	-332.314	α_{108}	-0.639
α_{23}	21.569	21.569	21.569	21.569	α_{66}	436.301	α_{109}	0.551
α_{24}	-7.010	-7.010	-7.010	-7.010	α_{67}	136.267	α_{110}	-0.134
α_{25}	23.803	23.803	23.803	23.803	α_{68}	-76.090	α_{111}	4.600
α_{26}	-11.156	-11.156	-11.156	-11.156	α_{69}	-20.368	α_{112}	-4.973
α_{27}	0.761	0.761	0.761	0.761	α_{70}	21.325	α_{113}	1.400
α_{28}	0.727	0.727	0.727	0.727	α_{71}	0.385	α_{114}	-1.605
α_{29}	7.192	7.192	7.192	7.192	α_{72}	0.870	α_{115}	-18.815
α_{30}	-9.265	-9.265	-9.265	-9.265	α_{73}	-6.663	α_{116}	6.464
α_{31}	-15.571	-15.571	-15.571	-15.571	α_{74}	-0.069	α_{117}	0.768
α_{32}	-2.696	-2.696	-2.696	-2.696	α_{75}	-0.435	α_{118}	17.957
α_{33}	-5.727	-5.727	-5.727	-5.727	α_{76}	0.860	α_{119}	-4.810
α_{34}	-40.120	-40.120	-40.120	-40.120	α_{77}	0.278	α_{120}	-0.189
α_{35}	14.078	14.078	14.078	14.078	α_{78}	-1.344	α_{121}	0.116
α_{36}	-10.979	-10.979	-10.979	-10.979	α_{79}	0.656	α_{122}	0.217
α_{37}	-14.151	-14.151	-14.151	-14.151	α_{80}	-1.360	α_{123}	-4.262
α_{38}	5.281	5.281	5.281	5.281	α_{81}	6.218	α_{124}	4.484
α_{39}	-6.229	-6.229	-6.229	-6.229	α_{82}	-2.228	α_{125}	0.179
α_{40}	0.688	0.688	0.688	0.688	α_{83}	-0.207	α_{126}	-0.463
α_{41}	17.165	17.165	17.165	17.165	α_{84}	-0.179	α_{127}	0.210
α_{42}	16.123	16.123	16.123	16.123	α_{85}	-2.158	α_{128}	-0.653

*Intercepts in all outcome models were modified so that the mean of Y remained as 0.

T A B L E S23 Parameter values (coefficients) used in the simulation study for the outcome (Complex-2 scenarios) (continued)

Parameters	**Complex-2**	Parameters	**Complex-2**	Parameters	**Complex-2**
	All n		All n		All n
α_{129}	-0.019	α_{173}	-0.003	α_{217}	0.0001
α_{130}	0.273	α_{174}	-0.466	α_{218}	0.0001
α_{131}	0.089	α_{175}	1.204	α_{219}	0.003
α_{132}	-0.673	α_{176}	0.077	α_{220}	-0.0005
α_{133}	0.330	α_{177}	0.083	α_{221}	-0.059
α_{134}	-0.032	α_{178}	-1.889	α_{222}	0.777
α_{135}	-3.182	α_{179}	-1.461	α_{223}	-0.037
α_{136}	-0.037	α_{180}	1.472	α_{224}	2.530
α_{137}	0.038	α_{181}	1.764	α_{225}	1.118
α_{138}	2.075	α_{182}	-0.044	α_{226}	-1.766
α_{139}	0.042	α_{183}	-0.051	α_{227}	1.109
α_{140}	-0.026	α_{184}	-0.012	α_{228}	0.014
α_{141}	-0.009	α_{185}	0.048	α_{229}	9.805
α_{142}	0.0001	α_{186}	0.0001	α_{230}	2.544
α_{143}	1.273	α_{187}	-0.033	α_{231}	-2.017
α_{144}	0.093	α_{188}	-0.003	α_{232}	-2.290
α_{145}	-0.061	α_{189}	-0.040	α_{233}	-13.214
α_{146}	0.005	α_{190}	0.088	α_{234}	15.207
α_{147}	-0.025	α_{191}	-4.076	α_{235}	1.587
α_{148}	0.002	α_{192}	-0.121	α_{236}	-11.014
α_{149}	-0.002	α_{193}	0.736	α_{237}	-0.537
α_{150}	0.001	α_{194}	-0.005	α_{238}	-0.181
α_{151}	0.002	α_{195}	1.750	α_{239}	-0.979
α_{152}	0.056	α_{196}	-0.036	α_{240}	-1.690
α_{153}	-0.040	α_{197}	-1.473	α_{241}	-0.168
α_{154}	0.049	α_{198}	-1.068		
α_{155}	0.0001	α_{199}	-0.154		
α_{156}	-0.001	α_{200}	-0.989		
α_{157}	0.049	α_{201}	0.001		
α_{158}	0.007	α_{202}	0.505		
α_{159}	-1.273	α_{203}	0.025		
α_{160}	0.0001	α_{204}	2.341		
α_{161}	-0.006	α_{205}	-0.796		
α_{162}	-0.082	α_{206}	0.180		
α_{163}	-0.034	α_{207}	0.0001		
α_{164}	0.036	α_{208}	0.119		
α_{165}	-3.915	α_{209}	0.008		
α_{166}	2.134	α_{210}	0.119		
α_{167}	1.643	α_{211}	0.087		
α_{168}	0.063	α_{212}	0.0001		
α_{169}	-0.082	α_{213}	-1.170		
α_{170}	-0.930	α_{214}	-0.032		
α_{171}	0.303	α_{215}	2.262		
α_{172}	-0.002	α_{216}	-0.051		

Section 5 | SUPER LEARNER CANDIDATE LIBRARIES AND SCREENING ALGORITHMS

In Super Learner, a (1) **reduced library** (non-data adaptive and adaptive parametric approaches), and (2) a **full library** (the reduced library in addition to non-parametric data-adaptive approaches) was considered:

TABLE S24 Overview of learners used within Super Learner

Learners	Reduced Library	Full Library
**a. Parametric (GLM) Library**	**(1) Reduced Library (a and b)**	**(2) Full Library (a, b and c)**
SL.mean (marginal mean of outcome, include as a benchmark)		
SL.glm (GLM)		
SL.glm.interaction (GLM with pairwise variable interactions)		
SL.bayesglm (Bayesian GLM)		
SL.gam (Generalised additive model)		
**b. Parametric, data-adaptive Library**		
SL.glmnet (GLM with Lasso/Elastic net regularisation)		
**c. Non-parametric, data-adaptive (ML) Library**		
SL.ranger (fast implementation random forest)		
SL.rpart (regression tree)		
SL.nnet (neural networks)		
SL.earth (multivariate adaptive regression splines)		
SL.xgboost (extreme gradient boosting)		
SL.ipregbragg (bagging tree)		
SL.svm (Support vector machines)		

Section 6 | TRUE VALUES ACE USED IN THE SIMULATION STUDY

TABLE S25 True values of the ACE for each scenario (data-generating mechanism and sample size), that were used when computing the performance measures. A large simulated dataset of size 1000000 was used to obtain the true values.

Mechanism	Sample size	Main effect of X	true ACE
Simple-1	200	0.45	0.45
	500	0.30	0.30
	1000	0.20	0.20
	2000	0.15	0.15
Complex-1a	200	-0.90	0.95
	500	-1.25	0.60
	1000	-1.50	0.35
	2000	-1.65	0.20
Complex-1b	200	-2.00	1.70
	500	-2.75	0.94
	1000	-3.13	0.58
	2000	-3.43	0.27
Simple-2	200	-0.65	-0.65
	500	-0.40	-0.40
	1000	-0.30	-0.30
	2000	-0.18	-0.18
Complex-2	200	1.70	5.08
	500	0.20	3.58
	1000	-1.40	1.98
	2000	-1.60	1.78

Section 7 | SIMULATION STUDY RESULTS

AIPW failed to produce sensible results for a few datasets (2-12 datasets across scenarios) when CF was applied. Specifically, the standard error was > 10 times the median standard error or the absolute value of the point estimate was > 5 times the absolute value of the median point estimate, where the median SE and point estimates were calculated using the results obtained across the 2000 datasets. These results were excluded from Figures 2-5, and further details regarding the scenarios and number of datasets affected, and the value of performance measures including and excluding results from the affected datasets are presented in Table S26. Full simulation results that include all datasets (n_{sim} = 2000) are presented in Tables S27 - S34.

TABLE S26 Scenarios, number of datasets affected, and performance measures including and excluding affected datasets

Mechanism	Sample size	Library	Folds used in CF	No. of datasets^	Relbias*	EmpSE*	RelModSE*	CP*
Simple-1	1000	Full	5	2	-1.21 [0.30]	0.12 [0.08]	87.52 [4.53]	95.82 [95.82]
Simple-1	2000	Reduced	2	5	2.30 [1.21]	0.07 [0.06]	59.50 [0.58]	95.09 [95.13]
Simple-2	2000	Full	10	4	1.22 [2.18]	0.14 [0.12]	72.41 [-41.76]	71.83 [71.65]
Complex-1a	2000	Full	2	9	-0.67 [-0.32]	0.16 [0.11]	39.50 [-3.55]	93.79 [93.77]
Simple-2	2000	Full	5	12	-10.69 [-6.62]	0.20 [0.12]	15.34 [-40.89]	71.14 [71.17]
Simple-2	2000	Full	10	5	-15.77 [-10.23]	0.31 [0.13]	-2.12 [-37.48]	71.82 [71.64]

^Number of affected datasets; *Performance measure including results from all datasets [performance measure excluding affected datasets in brackets].

TABLE S27 Simulation study results: Performance measures for the **Reduced library in SL** and sample size of **200** (n_{sim} = 2000)

Method	CF	Mechanism	Bias	Bias (MCSE)	RelBias	EmpSE	EmpSE (MCSE)	ModSE	ModSE (MCSE)	RelModSE	RelModSE (MCSE)	CP	CP (MCSE)
AIPW	No CF		0.0116	0.009	2.573	0.4070	0.006	0.42	0.00010	2.17	1.62	91.07	0.0064
TMLE	No CF		-0.0009	0.004	-0.194	0.1760	0.003	0.15	0.00010	-14.00	1.36	90.85	0.0064
AIPW	2-folds		0.0039	0.023	0.875	0.9950	0.016	1.17	0.00020	17.21	1.88	94.64	0.0051
TMLE	2-folds	Simple-1	0.0038	0.005	0.847	0.2260	0.004	0.22	0.00010	-2.28	1.54	95.05	0.0048
AIPW	5-folds		0.0033	0.017	0.734	0.7690	0.012	0.81	0.00020	5.17	1.69	95.66	0.0046
TMLE	5-folds		0.0014	0.004	0.309	0.1880	0.003	0.19	<0.0001	2.33	1.62	95.45	0.0047
AIPW	10-folds		0.0121	0.014	2.685	0.6360	0.010	0.73	0.00010	14.33	1.84	95.67	0.0046
TMLE	10-folds		0.0020	0.004	0.451	0.1830	0.003	0.19	<0.0001	2.77	1.62	95.85	0.0045
AIPW	No CF		-0.0790	0.010	-8.316	0.4470	0.007	0.33	0.00020	-26.59	1.16	85.69	0.0078
TMLE	No CF		-0.0862	0.008	-9.074	0.3570	0.006	0.27	0.00010	-25.03	1.19	85.45	0.0079
AIPW	2-folds		-0.0805	0.015	-8.470	0.6870	0.011	0.62	0.00020	-10.23	1.42	92.27	0.0060
TMLE	2-folds	Complex-1a	-0.0892	0.012	-9.385	0.5250	0.008	0.49	0.00010	-5.76	1.49	93.75	0.0054
AIPW	5-folds		-0.0678	0.015	-7.136	0.6550	0.010	0.61	0.00020	-6.89	1.49	94.51	0.0051
TMLE	5-folds		-0.0718	0.009	-7.562	0.4170	0.007	0.42	0.00010	1.59	1.61	94.25	0.0052
AIPW	10-folds		-0.0632	0.014	-6.653	0.6350	0.010	0.70	0.00010	10.15	1.77	95.31	0.0048
TMLE	10-folds		-0.0767	0.009	-8.077	0.3960	0.006	0.41	0.00010	3.02	1.63	94.85	0.0049
AIPW	No CF		-0.1520	0.017	-8.943	0.7550	0.012	0.56	0.00030	-25.36	1.18	82.79	0.0085
TMLE	No CF		-0.1714	0.015	-10.081	0.6770	0.011	0.50	0.00020	-26.43	1.16	82.75	0.0084
AIPW	2-folds		-0.1671	0.026	-9.829	1.1600	0.018	1.02	0.00030	-12.17	1.39	92.06	0.0061
TMLE	2-folds	Complex-1b	-0.1915	0.022	-11.265	1.0020	0.016	0.93	0.00030	-6.91	1.47	92.55	0.0059
AIPW	5-folds		-0.1331	0.022	-7.827	0.9800	0.016	0.97	0.00030	-0.79	1.59	93.05	0.0057
TMLE	5-folds		-0.1601	0.018	-9.418	0.8260	0.013	0.80	0.00020	-2.58	1.54	93.60	0.0055
AIPW	10-folds		-0.1912	0.023	-11.248	1.0340	0.017	1.01	0.00030	-2.06	1.57	93.90	0.0054
TMLE	10-folds		-0.1767	0.017	-10.394	0.7670	0.012	0.76	0.00020	-0.65	1.57	94.00	0.0053
AIPW	No CF		0.1486	0.017	-22.792	0.7470	0.012	0.30	0.00050	-59.95	0.64	57.31	0.0111
TMLE	No CF		0.1682	0.008	-25.800	0.3380	0.005	0.12	0.00020	-64.00	0.57	56.65	0.0111
AIPW	2-folds		0.1840	0.031	-28.230	1.3670	0.022	1.34	0.00040	-1.94	1.58	89.19	0.0071
TMLE	2-folds	Simple-2	0.1845	0.011	-28.306	0.4740	0.007	0.38	0.00010	-18.83	1.28	86.15	0.0077
AIPW	5-folds		0.1484	0.025	-22.767	1.1090	0.018	1.15	0.00030	3.90	1.66	89.86	0.0068
TMLE	5-folds		0.1532	0.009	-23.499	0.3970	0.006	0.32	0.00010	-19.97	1.27	85.55	0.0079
AIPW	10-folds		0.1389	0.029	-21.316	1.2800	0.021	1.20	0.00040	-6.02	1.51	90.20	0.0068
TMLE	10-folds		0.1419	0.008	-21.774	0.3720	0.006	0.31	0.00010	-16.81	1.32	86.40	0.0077
AIPW	No CF		-0.0607	0.089	-1.195	2.7160	0.063	1.69	0.00230	-37.84	1.44	72.93	0.0146
TMLE	No CF		-0.1613	0.090	-3.175	2.7390	0.064	1.72	0.00240	-37.33	1.46	72.87	0.0146
AIPW	2-folds		-0.9666	0.125	-19.027	3.5530	0.088	4.24	0.00180	19.23	2.96	96.55	0.0064
TMLE	2-folds	Complex-2	-0.6931	0.113	-13.644	3.2730	0.080	3.99	0.00160	21.84	2.97	97.51	0.0054
AIPW	5-folds		-0.7926	0.121	-15.602	3.4770	0.085	4.16	0.00170	19.53	2.93	96.39	0.0065
TMLE	5-folds		-0.6946	0.114	-13.673	3.3100	0.081	3.85	0.00170	16.25	2.84	96.54	0.0063
AIPW	10-folds		-0.6442	0.121	-12.681	3.5040	0.085	4.07	0.00180	16.12	2.83	95.97	0.0068
TMLE	10-folds		-0.7034	0.120	-13.847	3.4910	0.085	3.79	0.00190	8.52	2.64	95.76	0.0069

TABLE S28 Simulation study results: Performance measures for the **Full library in SL** and sample size of **200** (n_{sim} = 2000)

Method	CF	Mechanism	Bias	Bias (MCSE)	RelBias	EmpSE	EmpSE (MCSE)	ModSE	ModSE (MCSE)	RelModSE	RelModSE (MCSE)	CP	CP (MCSE)
AIPW	No CF		0.0160	0.007	3.548	0.3210	0.005	0.25	0.00010	-23.49	1.21	83.41	0.0083
TMLE	No CF		0.0016	0.004	0.346	0.1760	0.003	0.13	0.00010	-25.61	1.18	83.35	0.0083
AIPW	2-folds		-0.0080	0.017	-1.784	0.7460	0.012	0.84	0.00020	12.22	1.79	95.05	0.0049
TMLE	2-folds	Simple-1	-0.0009	0.005	-0.195	0.2250	0.004	0.24	0.00010	8.78	1.72	96.30	0.0042
AIPW	5-folds		-0.0211	0.015	-4.694	0.6710	0.011	0.67	0.00020	-0.05	1.60	95.96	0.0045
TMLE	5-folds		0.0008	0.004	0.176	0.1920	0.003	0.21	<0.0001	9.74	1.74	96.45	0.0041
AIPW	10-folds		0.0143	0.013	3.187	0.5750	0.009	0.65	0.00010	12.80	1.80	96.73	0.0040
TMLE	10-folds		0.0020	0.004	0.441	0.1890	0.003	0.20	<0.0001	8.35	1.71	96.55	0.0041
AIPW	No CF		-0.1319	0.009	-13.882	0.4150	0.007	0.26	0.00020	-38.08	0.98	77.84	0.0093
TMLE	No CF		-0.1343	0.008	-14.135	0.3570	0.006	0.24	0.00010	-33.34	1.05	77.55	0.0093
AIPW	2-folds		-0.1100	0.016	-11.579	0.6980	0.011	0.62	0.00020	-10.38	1.42	92.86	0.0058
TMLE	2-folds	Complex-1a	-0.1327	0.012	-13.971	0.5430	0.009	0.57	0.00010	5.50	1.67	94.35	0.0052
AIPW	5-folds		-0.0929	0.013	-9.779	0.5930	0.009	0.62	0.00010	3.90	1.66	95.41	0.0047
TMLE	5-folds		-0.1123	0.009	-11.825	0.4200	0.007	0.47	0.00010	10.97	1.75	95.65	0.0046
AIPW	10-folds		-0.1159	0.016	-12.198	0.6870	0.011	0.78	0.00020	14.19	1.83	95.38	0.0048
TMLE	10-folds		-0.1152	0.009	-12.121	0.3960	0.006	0.44	0.00010	12.26	1.78	95.30	0.0047
AIPW	No CF		-0.2576	0.017	-15.156	0.7510	0.012	0.54	0.00030	-28.78	1.13	76.49	0.0095
TMLE	No CF		-0.2561	0.015	-15.064	0.6730	0.011	0.45	0.00020	-32.70	1.06	76.09	0.0095
AIPW	2-folds		-0.2588	0.032	-15.225	1.4320	0.023	1.21	0.00040	-15.48	1.34	91.70	0.0062
TMLE	2-folds	Complex-1b	-0.2859	0.024	-16.816	1.0610	0.017	1.13	0.00030	6.06	1.68	94.00	0.0053
AIPW	5-folds		-0.2090	0.024	-12.293	1.0410	0.017	1.05	0.00030	0.81	1.61	94.24	0.0053
TMLE	5-folds		-0.2359	0.018	-13.876	0.8210	0.013	0.89	0.00020	8.73	1.72	94.90	0.0049
AIPW	10-folds		-0.2395	0.023	-14.090	1.0090	0.016	1.04	0.00030	2.67	1.64	94.56	0.0051
TMLE	10-folds		-0.2392	0.017	-14.068	0.7630	0.012	0.82	0.00020	8.02	1.71	94.90	0.0049
AIPW	No CF		0.1730	0.013	-26.545	0.5620	0.009	0.24	0.00030	-56.56	0.69	47.86	0.0112
TMLE	No CF		0.1857	0.006	-28.486	0.2880	0.005	0.11	0.00020	-62.35	0.60	47.90	0.0112
AIPW	2-folds		0.2371	0.020	-36.369	0.8780	0.014	0.78	0.00030	-11.24	1.41	87.02	0.0076
TMLE	2-folds	Simple-2	0.2002	0.009	-30.713	0.3910	0.006	0.34	0.00010	-14.27	1.36	84.45	0.0081
AIPW	5-folds		0.1925	0.018	-29.538	0.7860	0.012	0.76	0.00020	-3.56	1.53	87.56	0.0074
TMLE	5-folds		0.1679	0.008	-25.759	0.3430	0.005	0.28	0.00010	-17.56	1.30	83.65	0.0083
AIPW	10-folds		0.1560	0.020	-23.931	0.8760	0.014	0.93	0.00020	5.94	1.69	88.10	0.0073
TMLE	10-folds		0.1582	0.007	-24.264	0.3320	0.005	0.28	0.00010	-17.23	1.31	83.45	0.0083
AIPW	No CF		0.1847	0.064	3.636	1.4130	0.045	0.91	0.00220	-35.61	2.06	68.15	0.0210
TMLE	No CF		0.1394	0.064	2.745	1.4230	0.045	0.90	0.00230	-36.42	2.03	68.36	0.0209
AIPW	2-folds		-0.7910	0.150	-15.572	3.0990	0.106	3.41	0.00330	10.17	3.79	94.59	0.0110
TMLE	2-folds	Complex-2	-0.4459	0.133	-8.778	2.8000	0.094	3.19	0.00280	14.02	3.83	95.06	0.0103
AIPW	5-folds		-0.2118	0.107	-4.169	2.3620	0.076	2.55	0.00220	7.91	3.46	96.92	0.0078
TMLE	5-folds		-0.1171	0.097	-2.304	2.1480	0.069	2.29	0.00210	6.70	3.42	95.09	0.0098
AIPW	10-folds		-0.2400	0.108	-4.724	2.3750	0.077	2.56	0.00230	7.85	3.48	95.22	0.0097
TMLE	10-folds		-0.1500	0.101	-2.953	2.2150	0.071	2.31	0.00220	4.06	3.35	94.23	0.0106

TABLE S29 Simulation study results: Performance measures for the **Reduced library in SL** and sample size of **500** (n_{sim} = 2000)

Method	CF	Mechanism	Bias	Bias (MCSE)	RelBias	EmpSE	EmpSE (MCSE)	ModSE	ModSE (MCSE)	RelModSE	RelModSE (MCSE)	CP	CP (MCSE)
AIPW	No CF	Simple-1	0.0022	0.004	0.749	0.1580	0.002	0.19	<0.0001	20.72	1.91	92.43	0.0059
TMLE	No CF		-0.0005	0.002	-0.167	0.1100	0.002	0.10	<0.0001	-9.63	1.43	92.40	0.0059
AIPW	2-folds		0.0159	0.013	5.310	0.5920	0.009	0.81	0.00010	36.19	2.18	94.83	0.0050
TMLE	2-folds		0.0025	0.003	0.831	0.1240	0.002	0.12	<0.0001	-0.54	1.57	95.10	0.0048
AIPW	5-folds		0.0060	0.010	2.001	0.4250	0.007	0.44	0.00010	3.47	1.64	95.81	0.0045
TMLE	5-folds		0.0001	0.003	0.023	0.1130	0.002	0.11	<0.0001	-1.37	1.56	94.95	0.0049
AIPW	10-folds		0.0012	0.007	0.412	0.3140	0.005	0.35	0.00010	10.05	1.76	95.25	0.0048
TMLE	10-folds		0.0008	0.002	0.258	0.1110	0.002	0.11	<0.0001	-0.34	1.58	94.95	0.0049
AIPW	No CF	Complex-1a	-0.0469	0.005	-7.808	0.2390	0.004	0.18	0.00010	-23.80	1.21	87.56	0.0074
TMLE	No CF		-0.0506	0.004	-8.429	0.2010	0.003	0.16	0.00010	-17.60	1.30	87.70	0.0073
AIPW	2-folds		-0.0520	0.008	-8.660	0.3570	0.006	0.37	0.00010	3.22	1.64	93.69	0.0055
TMLE	2-folds		-0.0602	0.005	-10.041	0.2460	0.004	0.24	0.00010	-1.49	1.56	94.40	0.0051
AIPW	5-folds		-0.0395	0.005	-6.583	0.2380	0.004	0.28	0.00010	16.68	1.86	95.17	0.0048
TMLE	5-folds		-0.0427	0.005	-7.113	0.2170	0.003	0.23	0.00010	4.04	1.65	95.05	0.0049
AIPW	10-folds		-0.0260	0.006	-4.342	0.2550	0.004	0.26	0.00010	0.05	1.60	95.64	0.0046
TMLE	10-folds		-0.0324	0.005	-5.409	0.2140	0.003	0.22	0.00010	4.97	1.66	95.80	0.0045
AIPW	No CF	Complex-1b	-0.0575	0.011	-6.119	0.4780	0.008	0.36	0.00020	-25.31	1.18	88.60	0.0071
TMLE	No CF		-0.0664	0.008	-7.063	0.3510	0.006	0.30	0.00010	-15.24	1.34	89.05	0.0070
AIPW	2-folds		-0.1061	0.014	-11.289	0.6360	0.010	0.60	0.00020	-4.80	1.51	93.02	0.0057
TMLE	2-folds		-0.0993	0.011	-10.558	0.4910	0.008	0.45	0.00010	-7.59	1.46	93.05	0.0057
AIPW	5-folds		-0.0760	0.011	-8.079	0.4940	0.008	0.51	0.00010	3.50	1.65	95.23	0.0048
TMLE	5-folds		-0.0663	0.009	-7.049	0.4010	0.006	0.41	0.00010	2.48	1.62	95.10	0.0048
AIPW	10-folds		-0.0399	0.012	-4.247	0.5220	0.008	0.58	0.00010	10.86	1.77	96.06	0.0044
TMLE	10-folds		-0.0456	0.009	-4.847	0.3930	0.006	0.41	0.00010	4.74	1.66	96.25	0.0042
AIPW	No CF	Simple-2	0.0633	0.008	-16.038	0.3380	0.005	0.18	0.00020	-46.56	0.86	52.29	0.0113
TMLE	No CF		0.0603	0.005	-15.282	0.2360	0.004	0.07	0.00020	-68.64	0.50	49.80	0.0112
AIPW	2-folds		0.1032	0.028	-26.138	1.2140	0.019	1.16	0.00030	-4.32	1.53	83.17	0.0085
TMLE	2-folds		0.0994	0.009	-25.183	0.4110	0.006	0.26	0.00020	-35.45	1.02	80.85	0.0088
AIPW	5-folds		0.0529	0.015	-13.410	0.6300	0.011	0.62	0.00020	-2.05	1.70	86.54	0.0083
TMLE	5-folds		0.0600	0.006	-15.207	0.2710	0.004	0.19	0.00010	-28.71	1.13	82.50	0.0085
AIPW	10-folds		0.0382	0.016	-9.688	0.6320	0.011	0.62	0.00020	-1.99	1.74	86.93	0.0084
TMLE	10-folds		0.0528	0.006	-13.387	0.2670	0.004	0.19	0.00010	-29.44	1.12	82.50	0.0085
AIPW	No CF	Complex-2	0.3615	0.053	10.099	1.6650	0.038	0.99	0.00140	-40.78	1.34	72.13	0.0143
TMLE	No CF		0.3428	0.056	9.577	1.7690	0.040	1.02	0.00150	-42.14	1.30	70.58	0.0144
AIPW	2-folds		0.2489	0.080	6.952	2.5100	0.057	2.54	0.00130	1.02	2.28	93.50	0.0079
TMLE	2-folds		0.2214	0.080	6.185	2.5080	0.057	2.48	0.00130	-1.32	2.23	92.78	0.0082
AIPW	5-folds		0.3946	0.088	11.023	2.5300	0.062	2.52	0.00150	-0.55	2.46	93.30	0.0087
TMLE	5-folds		0.3892	0.084	10.870	2.6370	0.059	2.57	0.00140	-2.45	2.20	91.66	0.0088
AIPW	10-folds		0.3559	0.088	9.941	2.5320	0.062	2.45	0.00160	-3.42	2.38	93.45	0.0086
TMLE	10-folds		0.3060	0.084	8.547	2.6340	0.059	2.49	0.00140	-5.64	2.13	93.08	0.0081

TABLE S30 Simulation study results: Performance measures for the **Full library in SL** and sample size of **500** (n_{sim} = 2000)

Method	CF	Mechanism	Bias	Bias (MCSE)	RelBias	EmpSE	EmpSE (MCSE)	ModSE	ModSE (MCSE)	RelModSE	RelModSE (MCSE)	CP	CP (MCSE)
AIPW	No CF	Simple-1	-0.0005	0.003	-0.180	0.1190	0.002	0.12	<0.0001	-1.77	1.56	86.62	0.0076
TMLE	No CF		0.0006	0.002	0.182	0.1100	0.002	0.09	<0.0001	-19.80	1.27	86.85	0.0076
AIPW	2-folds		0.0066	0.008	2.186	0.3620	0.006	0.46	0.00010	27.03	2.02	94.73	0.0050
TMLE	2-folds		-0.0009	0.003	-0.310	0.1270	0.002	0.13	<0.0001	0.31	1.59	95.15	0.0048
AIPW	5-folds		0.0045	0.005	1.505	0.2330	0.004	0.30	<0.0001	30.86	2.08	95.15	0.0048
TMLE	5-folds		0.0022	0.003	0.745	0.1140	0.002	0.12	<0.0001	3.17	1.63	95.30	0.0047
AIPW	10-folds		-0.0000	0.004	-0.017	0.1960	0.003	0.21	<0.0001	4.85	1.67	95.59	0.0046
TMLE	10-folds		0.0029	0.003	0.964	0.1130	0.002	0.12	<0.0001	2.41	1.62	95.35	0.0047
AIPW	No CF	Complex-1a	-0.1014	0.007	-16.899	0.3020	0.005	0.17	0.00010	-42.52	0.91	75.69	0.0096
TMLE	No CF		-0.1028	0.004	-17.141	0.1950	0.003	0.14	0.00010	-30.91	1.09	76.45	0.0095
AIPW	2-folds		-0.0790	0.008	-13.164	0.3380	0.005	0.42	0.00010	24.12	1.97	93.09	0.0057
TMLE	2-folds		-0.0924	0.006	-15.394	0.2550	0.004	0.26	0.00010	3.00	1.63	93.95	0.0053
AIPW	5-folds		-0.0595	0.006	-9.913	0.2650	0.004	0.26	0.00010	0.16	1.59	95.33	0.0047
TMLE	5-folds		-0.0701	0.005	-11.681	0.2130	0.003	0.23	<0.0001	8.29	1.71	95.40	0.0047
AIPW	10-folds		-0.0406	0.009	-6.767	0.3850	0.006	0.40	0.00010	3.05	1.65	95.56	0.0047
TMLE	10-folds		-0.0596	0.005	-9.928	0.2070	0.003	0.23	<0.0001	9.48	1.73	95.60	0.0046
AIPW	No CF	Complex-1b	-0.1556	0.009	-16.550	0.4160	0.007	0.30	0.00010	-27.13	1.16	76.68	0.0095
TMLE	No CF		-0.1578	0.008	-16.788	0.3500	0.005	0.25	0.00010	-28.81	1.13	77.50	0.0093
AIPW	2-folds		-0.1477	0.015	-15.713	0.6450	0.010	0.56	0.00020	-13.32	1.38	92.00	0.0061
TMLE	2-folds		-0.1579	0.011	-16.795	0.4830	0.008	0.49	0.00010	0.95	1.60	93.00	0.0057
AIPW	5-folds		-0.1113	0.011	-11.840	0.4980	0.008	0.48	0.00010	-3.37	1.54	95.53	0.0047
TMLE	5-folds		-0.1168	0.009	-12.427	0.3890	0.006	0.42	0.00010	7.64	1.70	95.60	0.0046
AIPW	10-folds		-0.0796	0.011	-8.467	0.4970	0.008	0.54	0.00010	7.80	1.72	95.65	0.0046
TMLE	10-folds		-0.0972	0.008	-10.335	0.3790	0.006	0.41	0.00010	8.43	1.72	95.60	0.0046
AIPW	No CF	Simple-2	0.1018	0.006	-25.804	0.2790	0.004	0.10	0.00020	-63.32	0.59	45.71	0.0113
TMLE	No CF		0.0997	0.004	-25.259	0.1970	0.003	0.06	0.00020	-67.00	0.53	45.41	0.0112
AIPW	2-folds		0.1281	0.016	-32.463	0.6990	0.011	0.60	0.00020	-14.66	1.36	83.79	0.0083
TMLE	2-folds		0.1201	0.006	-30.441	0.2840	0.004	0.20	0.00010	-27.61	1.15	80.55	0.0089
AIPW	5-folds		0.0978	0.010	-24.792	0.4210	0.007	0.40	0.00010	-5.63	1.65	83.66	0.0091
TMLE	5-folds		0.0930	0.005	-23.553	0.2370	0.004	0.17	0.00010	-29.09	1.13	78.99	0.0091
AIPW	10-folds		0.0964	0.010	-24.414	0.3900	0.007	0.40	0.00010	2.18	1.83	82.07	0.0097
TMLE	10-folds		0.0839	0.005	-21.263	0.2370	0.004	0.16	0.00010	-30.27	1.13	78.77	0.0094
AIPW	No CF	Complex-2	0.2991	0.035	8.354	0.7710	0.025	0.42	0.00140	-44.95	1.77	62.30	0.0219
TMLE	No CF		0.2985	0.037	8.338	0.8340	0.026	0.57	0.00120	-31.88	2.16	65.33	0.0213
AIPW	2-folds		0.0790	0.059	2.205	1.3190	0.042	1.29	0.00140	-2.10	3.11	93.75	0.0109
TMLE	2-folds		0.0464	0.061	1.297	1.3640	0.043	1.29	0.00150	-5.51	3.00	92.15	0.0121
AIPW	5-folds		0.2737	0.058	7.645	1.1700	0.041	1.21	0.00140	3.24	3.65	93.28	0.0125
TMLE	5-folds		0.2944	0.063	8.225	1.4030	0.044	1.42	0.00140	1.43	3.21	91.38	0.0126
AIPW	10-folds		0.2182	0.051	6.094	1.0350	0.036	1.04	0.00120	0.53	3.51	92.23	0.0132
TMLE	10-folds		0.1870	0.057	5.224	1.2850	0.041	1.19	0.00140	-7.23	2.94	90.00	0.0134

TABLE S31 Simulation study results: Performance measures for the **Reduced library in SL** and sample size of **1000** (n_{sim} = 2000)

Method	CF	Mechanism	Bias	Bias (MCSE)	RelBias	EmpSE	EmpSE (MCSE)	ModSE	ModSE (MCSE)	RelModSE	RelModSE (MCSE)	CP	CP (MCSE)
AIPW	No CF	Simple-1	0.0009	0.002	0.469	0.0910	0.001	0.07	<0.0001	-19.12	1.28	94.24	0.0052
TMLE	No CF		-0.0002	0.002	-0.095	0.0740	0.001	0.07	<0.0001	-2.99	1.53	94.25	0.0052
AIPW	2-folds		0.0026	0.005	1.290	0.2030	0.003	0.27	<0.0001	32.67	2.11	94.91	0.0049
TMLE	2-folds		-0.0013	0.002	-0.629	0.0790	0.001	0.08	<0.0001	1.94	1.61	95.25	0.0048
AIPW	5-folds		0.0002	0.003	0.099	0.1420	0.002	0.18	<0.0001	26.96	2.01	95.78	0.0045
TMLE	5-folds		-0.0001	0.002	-0.068	0.0750	0.001	0.08	<0.0001	2.23	1.62	95.55	0.0046
AIPW	10-folds		-0.0052	0.005	-2.596	0.2340	0.004	0.22	0.00010	-4.14	1.52	95.87	0.0045
TMLE	10-folds		-0.0006	0.002	-0.280	0.0740	0.001	0.08	<0.0001	3.12	1.63	95.90	0.0044
AIPW	No CF	Complex-1a	0.0041	0.004	1.171	0.1720	0.003	0.13	0.00010	-26.60	1.16	89.24	0.0069
TMLE	No CF		0.0083	0.003	2.363	0.1350	0.002	0.11	<0.0001	-16.96	1.31	89.65	0.0068
AIPW	2-folds		-0.0288	0.005	-8.237	0.2080	0.003	0.19	0.00010	-9.46	1.44	93.49	0.0055
TMLE	2-folds		-0.0282	0.004	-8.053	0.1640	0.003	0.16	<0.0001	-3.44	1.53	93.85	0.0054
AIPW	5-folds		0.0065	0.005	1.857	0.2340	0.004	0.22	0.00010	-4.97	1.51	96.73	0.0040
TMLE	5-folds		0.0092	0.003	2.643	0.1430	0.002	0.15	<0.0001	5.59	1.67	96.35	0.0042
AIPW	10-folds		0.0128	0.005	3.646	0.2230	0.004	0.23	0.00010	2.32	1.62	96.12	0.0043
TMLE	10-folds		0.0152	0.003	4.330	0.1470	0.002	0.15	<0.0001	4.13	1.65	95.95	0.0044
AIPW	No CF	Complex-1b	0.0537	0.008	9.261	0.3660	0.006	0.25	0.00010	-31.93	1.08	88.63	0.0071
TMLE	No CF		0.0434	0.005	7.481	0.2350	0.004	0.19	0.00010	-17.44	1.31	89.35	0.0069
AIPW	2-folds		-0.0302	0.008	-5.215	0.3760	0.006	0.40	0.00010	4.93	1.66	93.47	0.0055
TMLE	2-folds		-0.0324	0.007	-5.580	0.2990	0.005	0.29	0.00010	-4.34	1.51	93.55	0.0055
AIPW	5-folds		0.0405	0.011	6.984	0.4740	0.007	0.50	0.00010	6.00	1.68	96.21	0.0043
TMLE	5-folds		0.0411	0.006	7.080	0.2720	0.004	0.28	0.00010	2.62	1.62	95.85	0.0045
AIPW	10-folds		0.0503	0.011	8.671	0.4970	0.008	0.54	0.00010	9.46	1.74	95.90	0.0045
TMLE	10-folds		0.0504	0.006	8.694	0.2690	0.004	0.28	0.00010	3.27	1.63	96.00	0.0044
AIPW	No CF	Simple-2	0.0206	0.005	-6.852	0.2170	0.003	0.06	0.00020	-72.10	0.45	52.73	0.0112
TMLE	No CF		0.0138	0.004	-4.591	0.1670	0.003	0.06	0.00010	-66.79	0.53	49.60	0.0112
AIPW	2-folds		0.0094	0.012	-3.140	0.5070	0.008	0.47	0.00010	-7.93	1.49	82.79	0.0086
TMLE	2-folds		0.0341	0.005	-11.360	0.2120	0.003	0.14	0.00010	-35.53	1.02	79.00	0.0091
AIPW	5-folds		0.0220	0.008	-7.321	0.3640	0.006	0.30	0.00010	-16.82	1.32	81.22	0.0088
TMLE	5-folds		0.0155	0.004	-5.177	0.1840	0.003	0.11	0.00010	-39.77	0.95	75.30	0.0096
AIPW	10-folds		0.0200	0.004	-6.656	0.1900	0.003	0.19	<0.0001	-2.30	1.55	79.57	0.0090
TMLE	10-folds		0.0136	0.004	-4.545	0.1830	0.003	0.11	0.00010	-41.47	0.93	74.90	0.0097
AIPW	No CF	Complex-2	0.3464	0.059	17.494	1.3270	0.042	0.75	0.00240	-43.59	1.80	69.54	0.0206
TMLE	No CF		0.3263	0.066	16.479	1.4840	0.047	0.73	0.00300	-50.88	1.57	64.13	0.0215
AIPW	2-folds		0.4492	0.090	22.689	1.9510	0.064	1.72	0.00230	-11.61	2.88	88.58	0.0146
TMLE	2-folds		0.4188	0.097	21.152	2.1720	0.069	1.85	0.00260	-14.60	2.71	86.55	0.0153
AIPW	5-folds		0.4412	0.086	22.283	1.9150	0.061	1.70	0.00220	-11.03	2.82	90.98	0.0128
TMLE	5-folds		0.4259	0.096	21.508	2.1410	0.068	1.65	0.00280	-22.84	2.45	85.60	0.0157
AIPW	10-folds		0.4370	0.082	22.070	1.8260	0.058	1.65	0.00200	-9.62	2.87	92.18	0.0120
TMLE	10-folds		0.3834	0.096	19.366	2.1400	0.068	1.69	0.00270	-21.14	2.50	86.77	0.0152

T A B L E S32 Simulation study results: Performance measures for the **Full library in SL** and sample size of **1000** (n_{sim} = 2000)

Method	CF	Mechanism	Bias	Bias (MCSE)	RelBias	EmpSE	EmpSE (MCSE)	ModSE	ModSE (MCSE)	RelModSE	RelModSE (MCSE)	CP	CP (MCSE)
AIPW	No CF	Simple-1	-0.0004	0.002	-0.203	0.0920	0.002	0.07	<0.0001	-27.05	1.16	90.69	0.0065
TMLE	No CF		0.0005	0.002	0.230	0.0740	0.001	0.06	<0.0001	-12.31	1.39	90.50	0.0066
AIPW	2-folds		0.0010	0.003	0.509	0.1120	0.002	0.14	<0.0001	27.02	2.02	95.52	0.0046
TMLE	2-folds		0.0005	0.002	0.258	0.0790	0.001	0.08	<0.0001	4.42	1.65	95.85	0.0045
AIPW	5-folds		-0.0022	0.003	-1.122	0.1200	0.002	0.23	<0.0001	87.52	2.98	95.82	0.0045
TMLE	5-folds		0.0005	0.002	0.257	0.0760	0.001	0.08	<0.0001	4.58	1.65	96.00	0.0044
AIPW	10-folds		0.0009	0.002	0.460	0.0810	0.001	0.08	<0.0001	2.13	1.62	96.32	0.0042
TMLE	10-folds		0.0004	0.002	0.212	0.0750	0.001	0.08	<0.0001	5.31	1.67	96.35	0.0042
AIPW	No CF	Complex-1a	-0.0619	0.005	-17.693	0.2220	0.004	0.11	0.00010	-50.06	0.79	76.30	0.0095
TMLE	No CF		-0.0509	0.003	-14.539	0.1320	0.002	0.09	<0.0001	-33.47	1.05	76.85	0.0094
AIPW	2-folds		-0.0603	0.005	-17.216	0.2010	0.003	0.17	0.00010	-13.43	1.38	92.29	0.0060
TMLE	2-folds		-0.0631	0.004	-18.017	0.1630	0.003	0.16	<0.0001	-2.61	1.54	92.50	0.0059
AIPW	5-folds		-0.0259	0.004	-7.393	0.1760	0.003	0.18	<0.0001	0.27	1.59	96.23	0.0043
TMLE	5-folds		-0.0261	0.003	-7.447	0.1380	0.002	0.15	<0.0001	6.33	1.68	96.25	0.0042
AIPW	10-folds		-0.0186	0.007	-5.321	0.3100	0.005	0.31	0.00010	0.54	1.60	96.47	0.0041
TMLE	10-folds		-0.0172	0.003	-4.925	0.1350	0.002	0.15	<0.0001	8.20	1.71	96.55	0.0041
AIPW	No CF	Complex-1b	-0.0696	0.008	-11.998	0.3550	0.006	0.19	0.00020	-45.86	0.86	80.27	0.0089
TMLE	No CF		-0.0621	0.005	-10.698	0.2280	0.004	0.16	0.00010	-29.91	1.11	81.90	0.0086
AIPW	2-folds		-0.0814	0.008	-14.033	0.3510	0.006	0.33	0.00010	-6.08	1.49	92.82	0.0058
TMLE	2-folds		-0.0898	0.006	-15.477	0.2830	0.004	0.28	0.00010	0.37	1.59	93.80	0.0054
AIPW	5-folds		-0.0348	0.008	-5.994	0.3690	0.006	0.46	0.00010	25.12	1.99	96.57	0.0041
TMLE	5-folds		-0.0356	0.006	-6.143	0.2460	0.004	0.27	0.00010	8.21	1.71	96.25	0.0042
AIPW	10-folds		-0.0102	0.011	-1.755	0.4840	0.008	0.47	0.00010	-2.05	1.56	95.51	0.0047
TMLE	10-folds		-0.0073	0.006	-1.264	0.2530	0.004	0.26	0.00010	3.03	1.63	95.70	0.0045
AIPW	No CF	Simple-2	0.0459	0.004	-15.314	0.1640	0.003	0.05	0.00010	-69.84	0.48	46.94	0.0112
TMLE	No CF		0.0417	0.003	-13.912	0.1520	0.002	0.05	0.00010	-68.64	0.50	46.50	0.0112
AIPW	2-folds		0.0689	0.008	-22.954	0.3530	0.006	0.26	0.00010	-27.77	1.17	79.81	0.0092
TMLE	2-folds		0.0700	0.004	-23.337	0.1760	0.003	0.12	0.00010	-33.08	1.06	76.65	0.0095
AIPW	5-folds		0.0472	0.005	-15.722	0.2260	0.004	0.23	0.00010	4.22	1.65	78.28	0.0092
TMLE	5-folds		0.0396	0.004	-13.184	0.1710	0.003	0.10	0.00010	-40.02	0.95	73.75	0.0098
AIPW	10-folds		0.0441	0.006	-14.703	0.2190	0.004	0.19	0.00010	-14.08	1.55	77.85	0.0106
TMLE	10-folds		0.0342	0.004	-11.415	0.1720	0.003	0.10	0.00010	-41.87	1.05	73.64	0.0112
AIPW	No CF	Complex-2	0.2557	0.023	12.913	0.5100	0.016	0.30	0.00090	-41.77	1.85	60.84	0.0219
TMLE	No CF		0.2247	0.023	11.349	0.5210	0.016	0.30	0.00090	-42.28	1.83	61.80	0.0217
AIPW	2-folds		0.1722	0.036	8.697	0.7760	0.025	0.71	0.00090	-8.23	2.99	92.83	0.0119
TMLE	2-folds		0.1178	0.046	5.949	1.0330	0.033	0.92	0.00120	-11.26	2.81	89.58	0.0137
AIPW	5-folds		0.1597	0.032	8.068	0.7050	0.022	0.67	0.00070	-4.83	3.02	90.34	0.0132
TMLE	5-folds		0.1132	0.039	5.715	0.8620	0.027	0.68	0.00110	-21.28	2.50	85.60	0.0157
AIPW	10-folds		0.1718	0.031	8.676	0.6850	0.022	0.66	0.00070	-3.25	3.07	90.96	0.0128
TMLE	10-folds		0.1349	0.038	6.814	0.8450	0.027	0.68	0.00110	-19.55	2.55	85.77	0.0156

TABLE S33 Simulation study results: Performance measures for the **Reduced library in SL** and sample size of **2000** (n_{sim} = 2000)

Method	CF	Mechanism	Bias	Bias (MCSE)	RelBias	EmpSE	EmpSE (MCSE)	ModSE	ModSE (MCSE)	RelModSE	RelModSE (MCSE)	CP	CP (MCSE)
AIPW	No CF	Simple-1	0.0013	0.001	0.850	0.0540	0.001	0.05	<0.0001	-4.31	1.51	93.60	0.0055
TMLE	No CF		0.0012	0.001	0.793	0.0540	0.001	0.05	<0.0001	-4.49	1.51	93.50	0.0055
AIPW	2-folds		0.0014	0.001	0.964	0.0630	0.001	0.08	<0.0001	19.78	1.90	95.29	0.0047
TMLE	2-folds		0.0007	0.001	0.465	0.0560	0.001	0.06	<0.0001	-1.50	1.56	95.10	0.0048
AIPW	5-folds		0.0013	0.002	0.880	0.0680	0.001	0.07	<0.0001	6.70	1.69	94.39	0.0051
TMLE	5-folds		0.0013	0.001	0.867	0.0550	0.001	0.05	<0.0001	-1.34	1.56	94.35	0.0052
AIPW	10-folds		0.0024	0.002	1.629	0.0830	0.001	0.07	<0.0001	-15.00	1.34	95.05	0.0049
TMLE	10-folds		0.0013	0.001	0.881	0.0540	0.001	0.05	<0.0001	-0.90	1.57	95.00	0.0049
AIPW	No CF	Complex-1a	0.0499	0.003	24.938	0.1300	0.002	0.09	<0.0001	-33.00	1.06	84.20	0.0082
TMLE	No CF		0.0545	0.002	27.259	0.0950	0.002	0.08	<0.0001	-16.79	1.32	84.05	0.0082
AIPW	2-folds		0.0222	0.006	11.116	0.2560	0.004	0.28	0.00010	10.55	1.75	93.69	0.0054
TMLE	2-folds		0.0275	0.003	13.731	0.1130	0.002	0.11	<0.0001	-4.25	1.51	93.95	0.0053
AIPW	5-folds		0.0554	0.008	27.681	0.3440	0.005	0.33	0.00010	-3.40	1.53	92.66	0.0058
TMLE	5-folds		0.0521	0.003	26.029	0.1160	0.002	0.12	<0.0001	-0.85	1.57	92.60	0.0059
AIPW	10-folds		0.0573	0.006	28.646	0.2730	0.004	0.27	0.00010	-0.41	1.58	92.91	0.0058
TMLE	10-folds		0.0577	0.003	28.854	0.1120	0.002	0.12	<0.0001	2.54	1.62	92.55	0.0059
AIPW	No CF	Complex-1b	0.1347	0.005	49.892	0.2110	0.003	0.14	0.00010	-35.22	1.02	75.00	0.0097
TMLE	No CF		0.1396	0.004	51.711	0.1580	0.002	0.13	<0.0001	-17.86	1.30	74.60	0.0097
AIPW	2-folds		0.0873	0.007	32.323	0.3060	0.005	0.29	0.00010	-5.92	1.49	91.37	0.0063
TMLE	2-folds		0.0884	0.005	32.731	0.2120	0.003	0.20	0.00010	-5.53	1.49	91.50	0.0062
AIPW	5-folds		0.1302	0.011	48.222	0.4720	0.007	0.52	0.00010	11.32	1.77	90.19	0.0067
TMLE	5-folds		0.1292	0.005	47.843	0.2160	0.003	0.21	0.00010	-2.23	1.55	89.35	0.0069
AIPW	10-folds		0.1461	0.009	54.100	0.4080	0.006	0.45	0.00010	10.91	1.76	88.87	0.0071
TMLE	10-folds		0.1478	0.005	54.746	0.2140	0.003	0.21	0.00010	-0.56	1.57	88.55	0.0071
AIPW	No CF	Simple-2	-0.0096	0.004	5.250	0.1800	0.003	0.06	0.00010	-66.86	0.53	54.24	0.0112
TMLE	No CF		-0.0060	0.003	3.267	0.1180	0.002	0.04	0.00010	-62.21	0.60	52.00	0.0112
AIPW	2-folds		0.0031	0.004	-1.682	0.1750	0.003	0.11	0.00010	-36.88	1.00	75.38	0.0096
TMLE	2-folds		0.0027	0.003	-1.509	0.1300	0.002	0.07	0.00010	-42.44	0.91	72.20	0.0100
AIPW	5-folds		0.0016	0.006	-0.875	0.2660	0.004	0.21	0.00010	-21.61	1.33	71.04	0.0109
TMLE	5-folds		-0.0041	0.003	2.250	0.1290	0.002	0.07	0.00010	-47.68	0.83	66.45	0.0106
AIPW	10-folds		-0.0022	0.005	1.219	0.1360	0.004	0.23	0.00010	72.41	4.83	71.83	0.0178
TMLE	10-folds		-0.0056	0.005	3.046	0.1260	0.003	0.07	0.00020	-47.25	1.35	68.90	0.0166
AIPW	No CF	Complex-2	0.3126	0.033	17.560	1.0280	0.023	0.64	0.00080	-38.19	1.40	72.66	0.0142
TMLE	No CF		0.2854	0.041	16.035	1.3040	0.029	0.68	0.00130	-47.82	1.17	64.19	0.0152
AIPW	2-folds		0.3795	0.041	21.320	1.2790	0.029	1.17	0.00070	-8.39	2.06	88.93	0.0100
TMLE	2-folds		0.3437	0.047	19.311	1.4770	0.033	1.14	0.00100	-22.50	1.74	83.70	0.0117
AIPW	5-folds		0.3851	0.045	21.632	1.3240	0.032	1.12	0.00090	-15.75	2.01	86.27	0.0116
TMLE	5-folds		0.3343	0.061	18.782	1.9250	0.043	1.40	0.00130	-27.08	1.64	78.95	0.0129
AIPW	10-folds		0.3399	0.049	19.095	1.4120	0.035	1.23	0.00100	-12.95	2.15	87.67	0.0115
TMLE	10-folds		0.3032	0.064	17.034	2.0190	0.045	1.41	0.00150	-30.11	1.57	81.15	0.0124

T A B L E S34 Simulation study results: Performance measures for the **Full library in SL** and sample size of **2000** (n_{sim} = 2000)

Method	CF	Mechanism	Bias	Bias (MCSE)	RelBias	EmpSE	EmpSE (MCSE)	ModSE	ModSE (MCSE)	RelModSE	RelModSE (MCSE)	CP	CP (MCSE)
AIPW	No CF		0.0014	0.001	0.922	0.0550	0.001	0.05	<0.0001	-13.50	1.37	91.00	0.0064
TMLE	No CF		0.0015	0.001	1.015	0.0540	0.001	0.05	<0.0001	-12.13	1.39	90.95	0.0064
AIPW	2-folds		0.0034	0.002	2.297	0.0690	0.001	0.11	<0.0001	59.50	2.52	95.09	0.0048
TMLE	2-folds	Simple-1	0.0017	0.001	1.132	0.0560	0.001	0.06	<0.0001	0.88	1.60	95.10	0.0048
AIPW	5-folds		0.0015	0.001	1.003	0.0550	0.001	0.06	<0.0001	-0.21	1.58	95.04	0.0049
TMLE	5-folds		0.0018	0.001	1.165	0.0550	0.001	0.06	<0.0001	0.20	1.58	95.15	0.0048
AIPW	10-folds		0.0032	0.002	2.167	0.0710	0.001	0.06	<0.0001	-8.82	1.44	95.54	0.0046
TMLE	10-folds		0.0021	0.001	1.375	0.0540	0.001	0.06	<0.0001	1.06	1.60	95.75	0.0045
AIPW	No CF		-0.0177	0.002	-8.851	0.1080	0.002	0.06	<0.0001	-41.19	0.93	80.95	0.0088
TMLE	No CF		-0.0109	0.002	-5.471	0.0900	0.001	0.06	<0.0001	-31.83	1.08	81.40	0.0087
AIPW	2-folds		-0.0013	0.004	-0.668	0.1590	0.002	0.22	<0.0001	39.50	2.21	93.79	0.0054
TMLE	2-folds	Complex-1a	-0.0031	0.002	-1.544	0.1050	0.002	0.10	<0.0001	-2.09	1.55	93.90	0.0054
AIPW	5-folds		0.0257	0.004	12.874	0.1790	0.003	0.18	<0.0001	0.58	1.59	95.08	0.0048
TMLE	5-folds		0.0196	0.002	9.807	0.1000	0.002	0.10	<0.0001	1.42	1.60	95.25	0.0048
AIPW	10-folds		0.0278	0.004	13.894	0.1720	0.003	0.15	<0.0001	-11.43	1.40	94.52	0.0051
TMLE	10-folds		0.0261	0.002	13.061	0.0970	0.002	0.10	<0.0001	2.82	1.63	94.30	0.0052
AIPW	No CF		0.0025	0.005	0.929	0.2040	0.003	0.11	0.00010	-44.44	0.88	82.58	0.0085
TMLE	No CF		0.0102	0.004	3.792	0.1580	0.002	0.11	0.00010	-31.64	1.08	82.90	0.0084
AIPW	2-folds		0.0050	0.009	1.845	0.4200	0.007	0.40	0.00010	-3.93	1.52	93.53	0.0055
TMLE	2-folds	Complex-1b	0.0193	0.004	7.166	0.1840	0.003	0.18	<0.0001	-0.96	1.57	93.85	0.0054
AIPW	5-folds		0.0703	0.007	26.021	0.3050	0.005	0.44	0.00010	45.09	2.30	94.17	0.0053
TMLE	5-folds		0.0613	0.004	22.695	0.1790	0.003	0.18	<0.0001	0.03	1.58	94.30	0.0052
AIPW	10-folds		0.0706	0.006	26.140	0.2660	0.004	0.29	0.00010	10.41	1.75	93.51	0.0055
TMLE	10-folds		0.0710	0.004	26.290	0.1700	0.003	0.18	<0.0001	3.06	1.63	93.65	0.0055
AIPW	No CF		0.0127	0.002	-6.977	0.1100	0.002	0.04	0.00010	-64.47	0.57	48.08	0.0112
TMLE	No CF		0.0105	0.002	-5.752	0.1110	0.002	0.04	0.00010	-65.52	0.55	47.85	0.0112
AIPW	2-folds		0.0271	0.003	-14.877	0.1200	0.002	0.07	<0.0001	-37.98	0.98	74.40	0.0098
TMLE	2-folds	Simple-2	0.0217	0.003	-11.900	0.1230	0.002	0.07	0.00010	-43.37	0.90	70.65	0.0102
AIPW	5-folds		0.0195	0.005	-10.691	0.2050	0.004	0.24	0.00010	15.34	1.95	71.14	0.0108
TMLE	5-folds		0.0103	0.003	-5.664	0.1230	0.002	0.06	0.00010	-47.86	0.83	67.05	0.0105
AIPW	10-folds		0.0287	0.011	-15.765	0.3120	0.008	0.31	0.00020	-2.12	2.51	71.82	0.0163
TMLE	10-folds		0.0117	0.004	-6.417	0.1250	0.003	0.06	0.00010	-49.07	1.19	67.99	0.0153
AIPW	No CF		0.2631	0.012	14.779	0.3900	0.009	0.26	0.00030	-34.48	1.47	62.47	0.0154
TMLE	No CF		0.2474	0.017	13.901	0.5310	0.012	0.29	0.00050	-44.59	1.24	61.00	0.0154
AIPW	2-folds		0.2096	0.018	11.775	0.5730	0.013	0.53	0.00030	-7.12	2.08	87.34	0.0105
TMLE	2-folds	Complex-2	0.1500	0.021	8.428	0.6650	0.015	0.47	0.00050	-29.53	1.58	81.58	0.0123
AIPW	5-folds		0.2161	0.021	12.142	0.4800	0.015	0.43	0.00050	-9.52	2.79	86.93	0.0147
TMLE	5-folds		0.1339	0.050	7.524	1.2150	0.035	0.93	0.00130	-23.52	2.22	80.37	0.0163
AIPW	10-folds		0.2170	0.027	12.191	0.5490	0.019	0.57	0.00060	3.70	3.61	86.99	0.0165
TMLE	10-folds		0.1803	0.062	10.130	1.3700	0.044	0.93	0.00200	-32.06	2.17	79.80	0.0180

Section 8 | FURTHER RESULTS: APPLICATION TO THE BIS CASE STUDY

TABLE S35 BIS case study results

Method	Library	CF used	Partially adjusted* (small confounder set)	Fully adjusted# (large confounder set)
AIPW	Reduced	No CF	1.050 (-0.063,2.163)	0.401 (-0.044,0.846)
TMLE			0.029 (-0.273,0.331)	0.554 (0.179,0.928)
AIPW	Full	No CF	-0.007 (-0.260,0.245)	0.011 (-0.388,0.409)
TMLE			0.026 (-0.217,0.270)	0.170 (-0.169,0.509)
AIPW	Reduced	2-folds	-0.037 (-0.570,0.496)	0.408 (-0.039,0.854)
TMLE			-0.004 (-0.532,0.524)	0.560 (0.184,0.936)
AIPW	Full	2-folds	-0.002 (-0.534,0.531)	0.063 (-0.413,0.538)
TMLE			0.030 (-0.499,0.558)	0.061 (-0.412,0.533)
AIPW	Reduced	5-folds	-0.072 (-0.597,0.452)	0.394 (-0.051,0.838)
TMLE			-0.037 (-0.556,0.481)	0.549 (0.174,0.924)
AIPW	Full	5-folds	0.095 (-0.777,0.967)	0.136 (-0.944,1.217)
TMLE			0.113 (-0.753,0.978)	0.102 (-0.577,0.780)
AIPW	Reduced	10-folds	0.044 (-0.477,0.565)	0.412 (-0.035,0.858)
TMLE			0.072 (-0.445,0.589)	0.564 (0.188,0.940)
AIPW	Full	10-folds	0.282 (-0.483,1.047)	-3.702 (-17.178,9.774)
TMLE			0.268 (-0.476,1.012)	0.079 (-0.487,0.645)

Results displayed are the estimated average causal effect (ACE) with accompanying 95% CI, of inflammation (GlycA) in 1-year old infants on pulse wave velocity (PWV) at 4 years of age (standardised), obtained by applying the methods to the BIS motivating example.
* Adjusted for demographic and background factors (i.e. excluding metabolomic measures).
# Adjusted for demographic and metabolites.

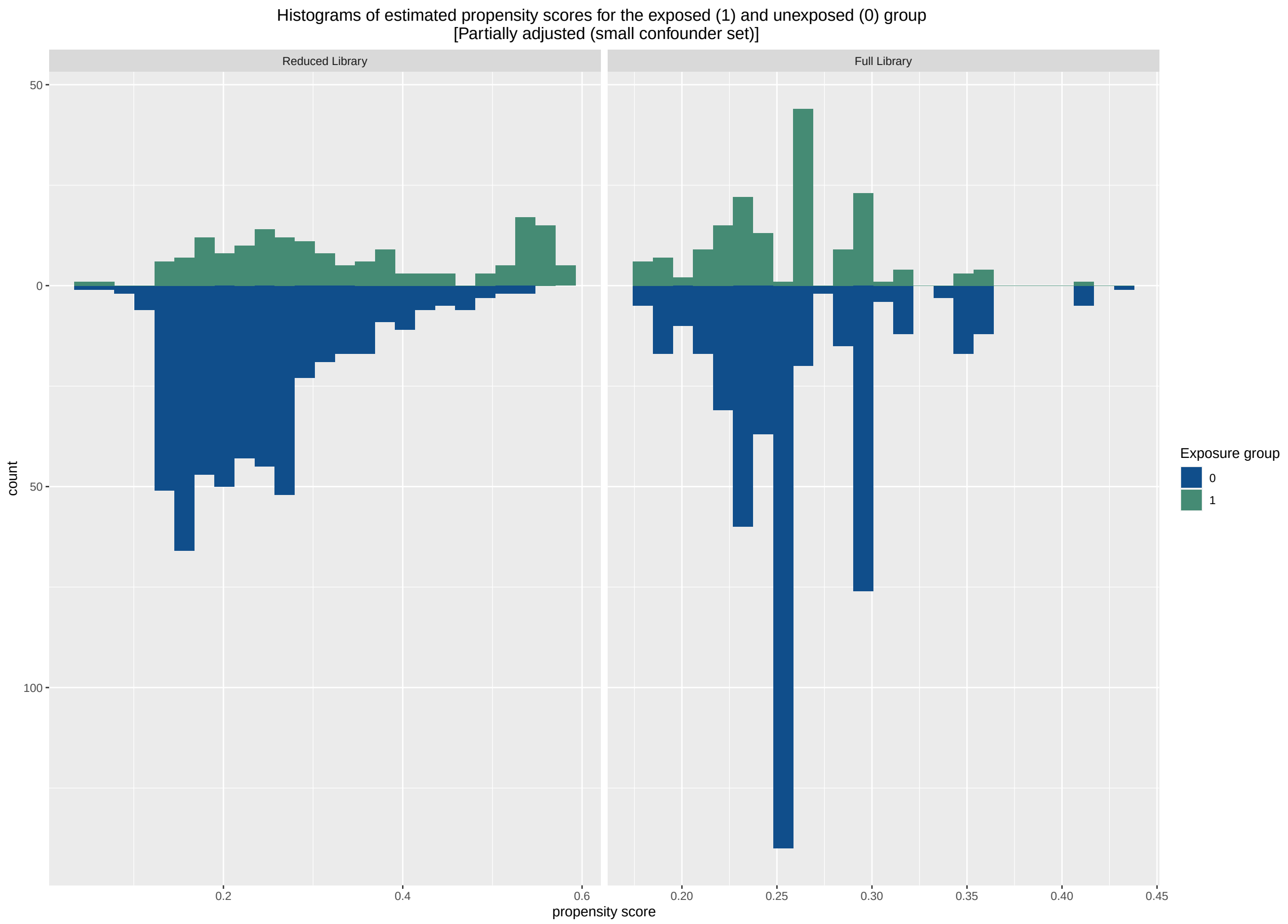

FIGURE S1 Distribution of estimated propensity scores for the BIS case study by exposure group (exposed and unexposed), for the partially adjusted (small confounder set).

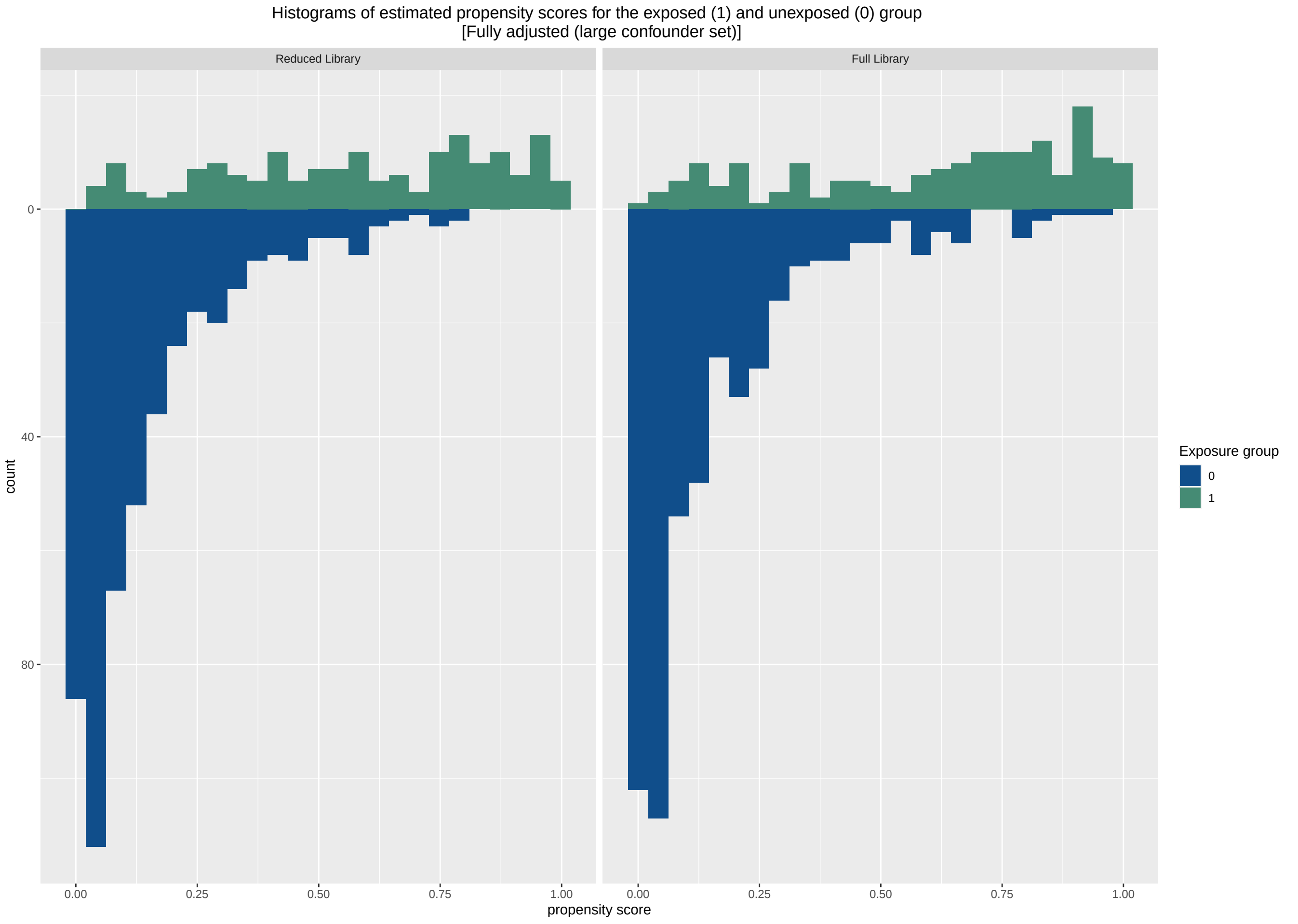

FIGURE S2 Distribution of estimated propensity scores for the BIS case study by exposure group (exposed and unexposed), for the fully adjusted (large confounder set).

Section 9 | FURTHER DETAILS: SL COEFFICIENTS AND PROPENSITY SCORES

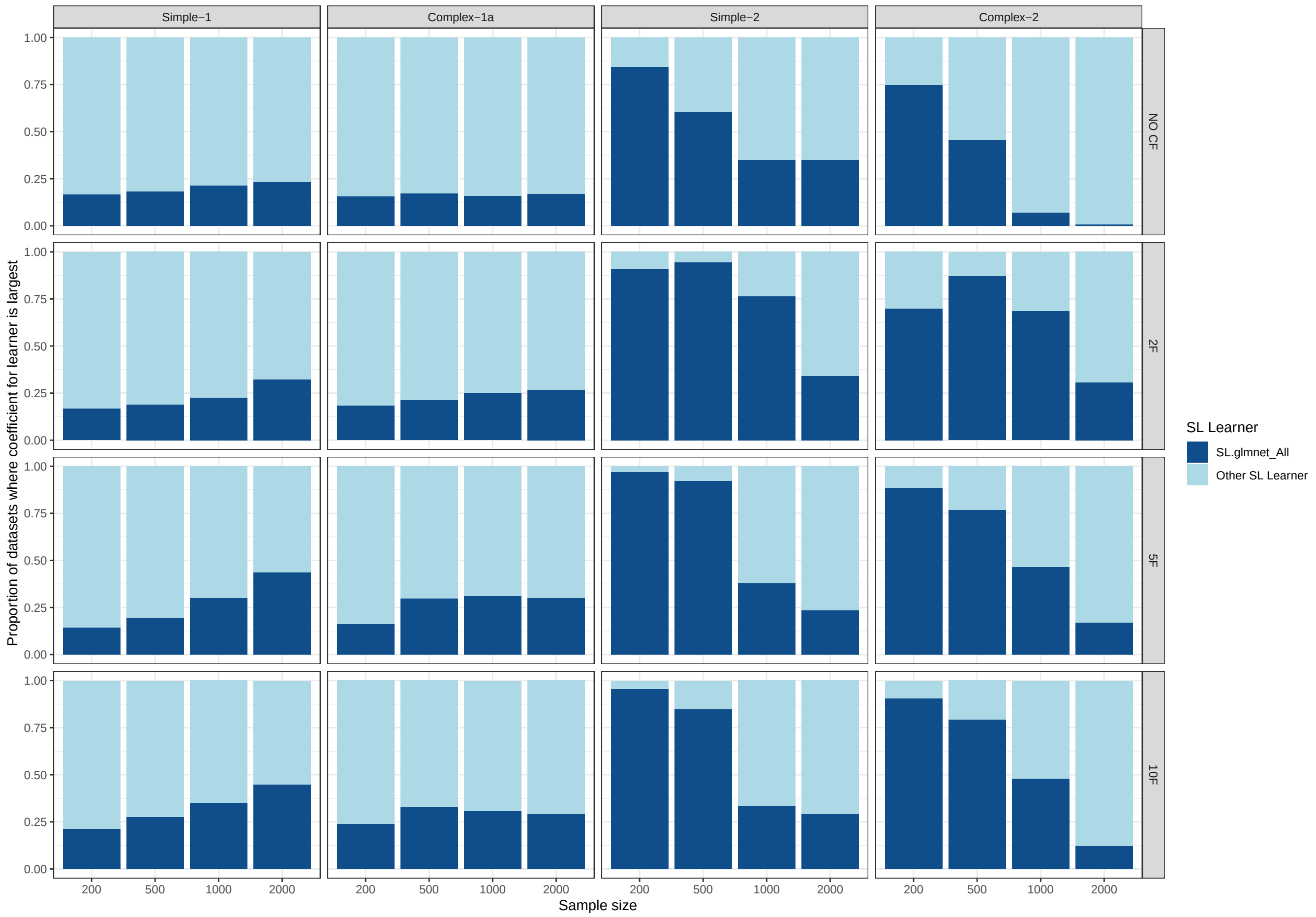

FIGURE S3 Summary of Super Learner (SL) coefficients for the Reduced Library when using SL to obtain estimated propensity scores, across scenarios (sample size and data-generating mechanism), without and with cross-fitting (2, 5, 10 folds). Dark blue shading refers the the proportion of datasets where the glmnet Learner had the largest coefficient. Lighter blue shading indicates the proportion of datasets where a learner other than glmnet had the largest coefficient.

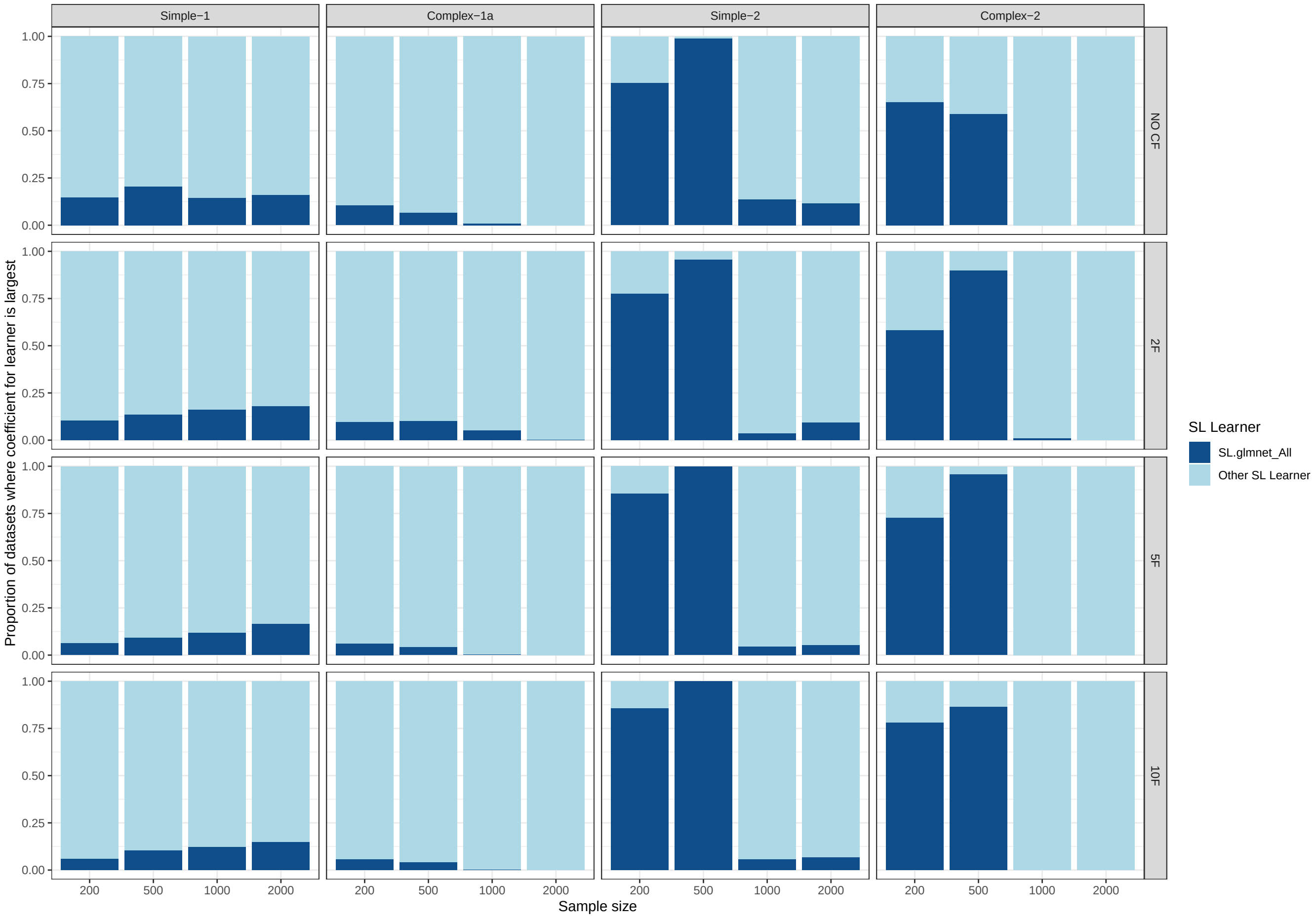

FIGURE S4 Summary of Super Learner (SL) coefficients for the Full Library when using SL to obtain estimated propensity scores, across scenarios (sample size and data-generating mechanism), without and with cross-fitting (2, 5, 10 folds). Dark blue shading refers the the proportion of datasets where the glmnet Learner had the largest coefficient. Lighter blue shading indicates the proportion of datasets where a learner other than glmnet had the largest coefficient.

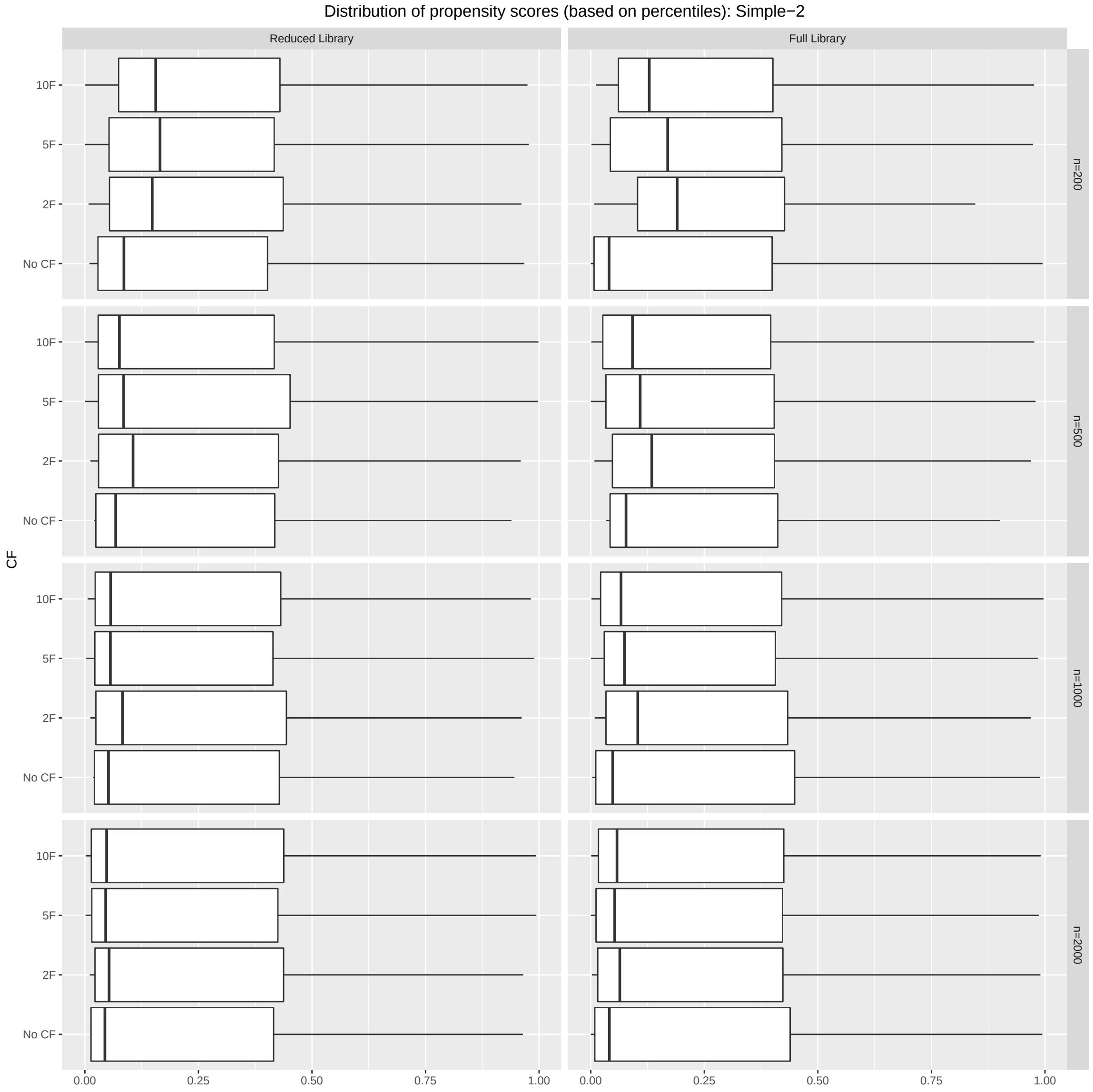

FIGURE S5 Example of the propensity score distribution for Simple-2 by library and sample size, without and with cross-fitting (2,5,10 folds). Box plots in the figure were plotted based on given percentiles of estimated propensity scores.

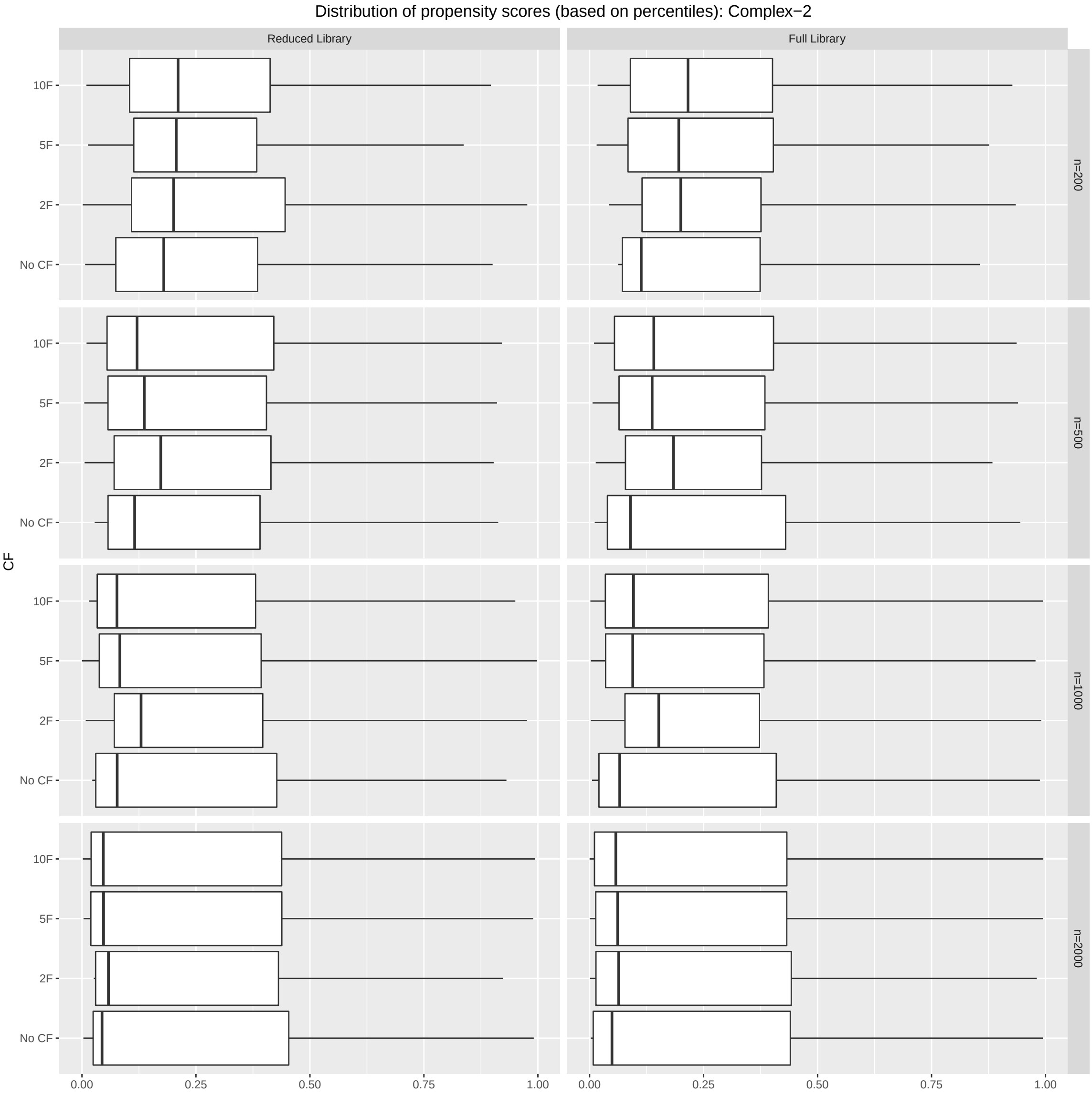

FIGURE S6 Example of the propensity score distribution for Complex-2 by library and sample size, without and with cross-fitting (2,5,10 folds). Box plots in the figure were plotted based on given percentiles of estimated propensity scores.

Section 10 | FURTHER EXPLORATION: SENSITIVITY AND DEPENDENCE ON SEEDS IN CROSS-FITTING

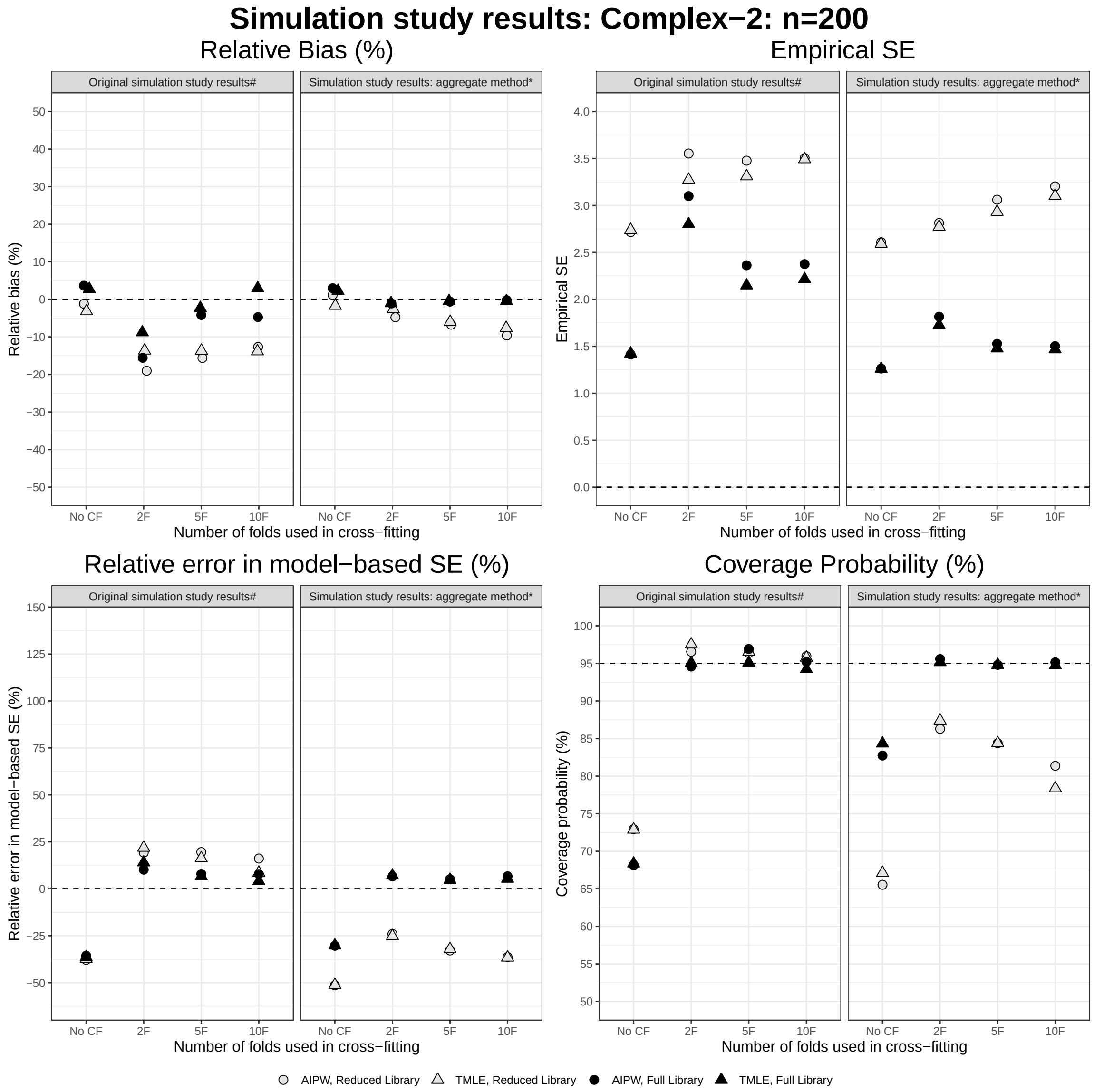

FIGURE S7 Simulation study results for the relative bias of point estimates (%), Empirical SE, bias in model-based SE (%), and coverage probability (%) for AIPW and TMLE with varying use of cross-fitting for complex-2 with sample size of n=200 (and using 2000 simulated datasets). #Original simulation study results reported in the main paper. *Results provided are based on 50 replications (different sample splits), where estimates for the methods were obtained across the replications using the median method (Chernozhukov et al.[22]). I.e. point estimates were obtained using $\tilde{\theta}_0^{median} = \text{median}\{\tilde{\theta}_0^s\}_{s=1}^{50}$ and variance obtained using $\hat{\sigma}^{2,median} = \text{median}\{\hat{\sigma}_s^2 + ((\hat{\sigma}_s - \tilde{\theta}_{median})(\hat{\sigma}_s - \tilde{\theta}_{median})')\}_{s=1}^{50}$, where $\tilde{\theta}_0^s$ and $\hat{\sigma}_s^2$ represent the point and variance estimates obtained for sample split $s\,(s = 1, ..., 50)$ respectively. The performance measures were then calculated as usual.

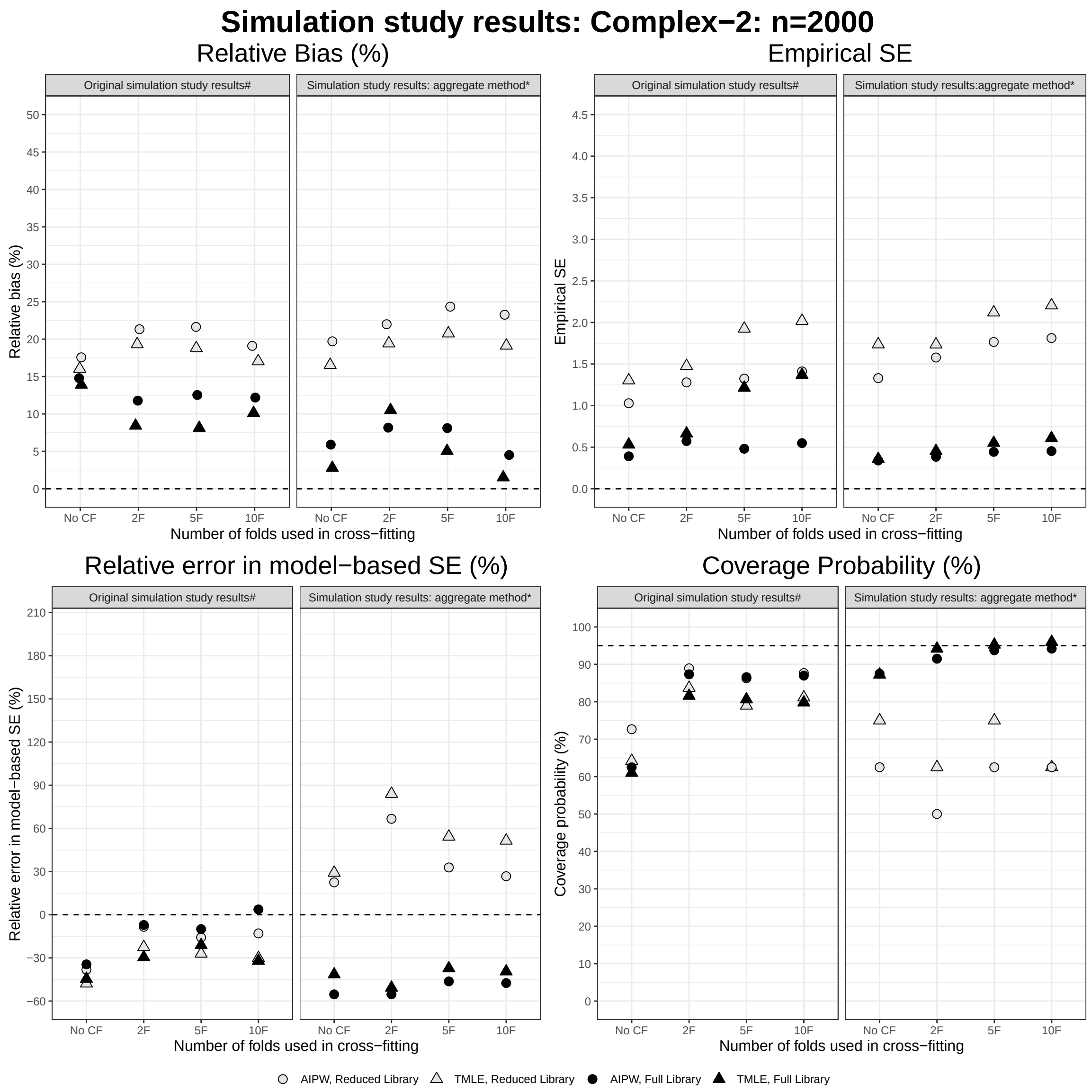

FIGURE S8 Simulation study results for the relative bias of point estimates (%), Empirical SE, bias in model-based SE (%), and coverage probability (%) for AIPW and TMLE with varying use of cross-fitting for complex-2 with sample size of n=2000 (and using 2000 simulated datasets). #Original simulation study results reported in the main paper. *Results provided are based on 50 replications (different sample splits), where estimates for the methods were obtained across the replications using the median method (Chernozhukov et al.[22]). I.e. point estimates were obtained using $\tilde{\theta}_0^{median} = \text{median}\{\tilde{\theta}_0^s\}_{s=1}^{50}$ and variance obtained using $\hat{\sigma}^{2,median} = \text{median}\{\hat{\sigma}_s^2 + ((\hat{\sigma}_s - \tilde{\theta}_{median})(\hat{\sigma}_s - \tilde{\theta}_{median})')\}_{s=1}^{50}$, where $\tilde{\theta}_0^s$ and $\hat{\sigma}_s^2$ represent the point and variance estimates obtained for sample split s ($s = 1, ..., 50$) respectively. The performance measures were then calculated as usual.

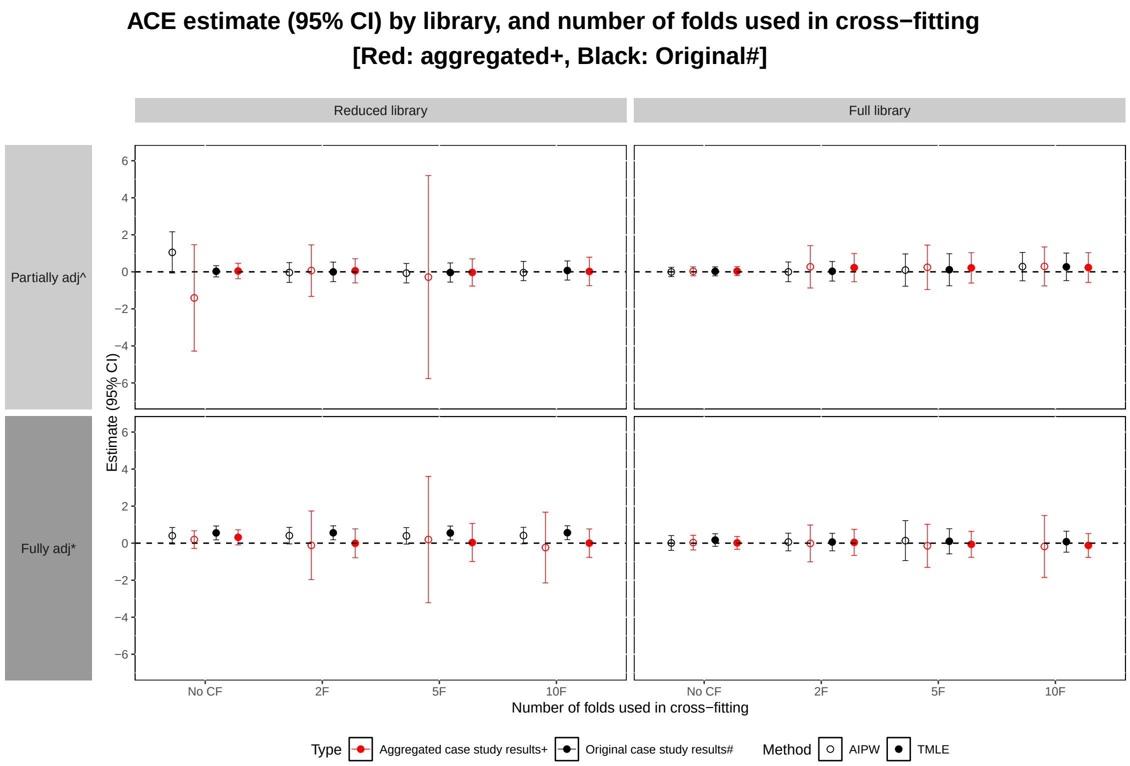

FIGURE S9 Estimated average causal effect (ACE) with accompanying 95% CI, of inflammation (GlycA) in 1-year old infants on Pulse Wave velocity (PWV) at 4 years of age (standardised), obtained by applying the methods to the BIS motivating example. ^demographic and background confounders only; *demographic, background, and metabolomic confounders.
Note: Original estimate for AIPW using fully adjusted confounder set and full library in SL with 10 fold cross-fitting not shown in figure as too large to be sensible (refer to Table 35 in Section 8 of the Supporting Information). #Original results are presented in black. +Results presented in red are based on 50 replications (different sample splits), where estimates for the methods were obtained across the replications using the median method (Chernozhukov et al.[22]). I.e. point estimates were obtained using $\tilde{\theta}_0^{median} = \text{median}\{\tilde{\theta}_0^s\}_{s=1}^{50}$ and variance obtained using $\hat{\sigma}^{2,median} = \text{median}\{\hat{\sigma}_s^2 + ((\hat{\sigma}_s - \tilde{\theta}_{median})(\hat{\sigma}_s - \tilde{\theta}_{median})')\}_{s=1}^{50}$, where $\tilde{\theta}_0^s$ and $\hat{\sigma}_s^2$ represent the point and variance estimates obtained for sample split s $(s = 1, ..., 50)$ respectively.
